# Anti-inflammatory Activity of Tanshinone-Related Diterpenes from *Perovskia artemisioides* Roots

**DOI:** 10.1021/acs.jnatprod.2c01004

**Published:** 2023-04-11

**Authors:** Zahra Sadeghi, Antonietta Cerulli, Stefania Marzocco, Mahdi Moridi Farimani, Milena Masullo, Sonia Piacente

**Affiliations:** † Department of Phytochemistry, Medicinal Plants and Drugs Research Institute, Shahid Beheshti University, Evin, Tehran 1983969411, Iran; ‡ Dipartimento di Farmacia, Università degli Studi di Salerno, via Giovanni Paolo II n. 132, 84084 Fisciano, Salerno, Italy

## Abstract

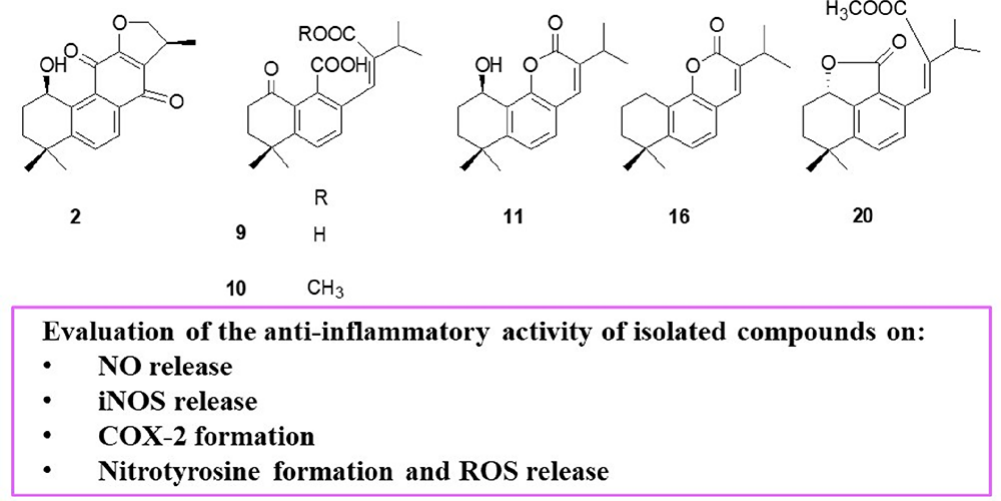

*Perovskia artemisioides* is a perennial and aromatic plant widely distributed in the Baluchestan region of Iran. Phytochemical analysis of a *n*-hexane extract of *P. artemisioides* roots, guided by an analytical approach based on LC-ESI/LTQOrbitrap/MS/MS, yielded six previously undescribed diterpenoid compounds (**2**, **9**–**11**, **16**, and **20**), and 19 known diterpenoids, for which the structures were elucidated by 1D and 2D NMR experiments. Some of the isolated compounds showed significant anti-inflammatory activity using J774A.1 macrophage cells stimulated with *Escherichia coli* lipopolysaccharide. In particular, compounds **6**, **8**, **17**, **18**, **20**, and **22** significantly inhibited the release of nitric oxide and the expression of related pro-inflammatory enzymes, such as inducible nitric oxide synthase and cycloxygenase-2. Moreover, two compounds that showed the highest activity in reducing nitric oxide release (**6** and **18**) were tested to evaluate their effects on nitrotyrosine formation and reactive oxygen species release. Both compounds inhibited ROS release and, in particular, compound **6** also inhibited nitrotyrosine formation at all tested concentrations, thus indicating a significant antioxidant potential.

*Perovskia* is a small genus of the family Lamiaceae, comprising nine species distributed mainly in the rocky areas of central Asia.^1^ In traditional medicine, extracts of *Perovskia* species are used as remedies for their antibacterial, anti-inflammatory, stomachic, expectorant, and carminative activities.^2^ Previous phytochemical investigations resulted in the occurrence of diterpenoid derivatives belonging to the abietane, icetexane, and isopimarane classes.^1,3^ Among the isolated abietanes, tanshinones are 20-norditerpenes with an abietane-type skeleton characterized by a phenanthrenequinone core, including *ortho*-quinone and *para*-quinone derivatives.^4^ Tanshinones were obtained mainly from *Salvia miltiorrhizae* roots, along with a few other genera from the Lamiaceae family, such as *Perovskia.*(5) Tanshinones show a wide variety of biological activities, including antioxidant, antidiabetic, and anti-inflammatory effects,^1^ and, in the past few years, they have attracted great attention for their cytotoxic properties and activity against cardiovascular and cerebrovascular diseases.^4^

The essential oil of *P. artemisioides* Boiss. (Lamiaceae) was investigated, and its antimicrobial and insecticidal activity were also reported.^6,7^ Our previous investigation focused on the chemical composition of *P. artemisioides* aerial parts. The extracts and isolated compounds belonging to the terpenoid and flavonoid classes showed excellent capabilities in inhibiting the formation of biofilms by different Gram-positive and Gram-negative pathogens.^2^

Herein, the phytochemical investigation of the *n-*hexane extract of *P. artemisioides* roots was performed, guided by an analytical approach based on LC-ESI/LTQOrbitrap/MS/MS. In this manner, six diterpenoid compounds (**2**, **9**–**11**, **16**, and **20**) not reported before in the literature, along with 19 known diterpenoids, were isolated and elucidated unambiguously by 1D and 2D NMR analysis. Considering the anti-inflammatory activity reported for tanshinone derivatives, the ability of diterpenoids isolated from *P. artemisioides* roots to inhibit the release of nitric oxide and the expression of related pro-inflammatory enzymes, such as inducible nitric oxide synthase and COX-2, was evaluated in a LPS-stimulated J774A.1 macrophage cell line. Furthermore, those compounds with the most potent activity in reducing NO release were also tested for their antioxidant potential.

## Results and Discussion

### LC-ESI/LTQOrbitrap/MS/MS Analysis of a *P. artemisioides* Root *n-*Hexane Extract

**Figure 1:**
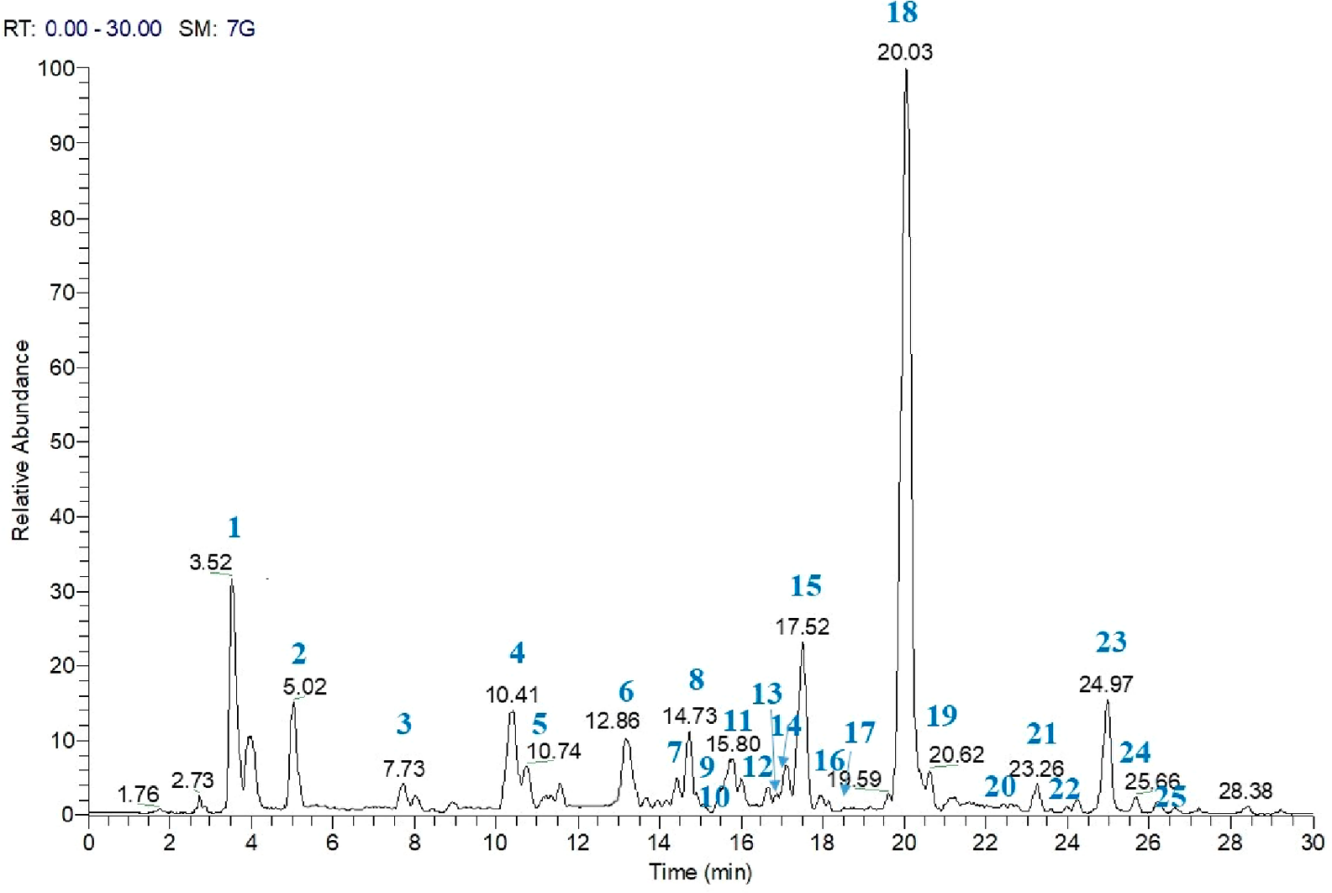
LC-MS analysis of the *n*-hexane extract of *P. artemisioides* roots in the positive-ion mode.

To identify the specialized metabolites occurring in the *n-*hexane extract of *P. artemisioides* roots, an analytical approach based on LC-(−)ESI/LTQOrbitrap/MS/MS was carried out (Figure 1). This procedure allowed the assignment of the [M + H]^+^ ions occurring in the LC-MS profile, leading to each molecular formula, by measuring the accurate molecular mass, and each putative structural identity, by careful analysis of the fragmentation pattern yielded in LC-MS/MS experiments.^8,9^ The LC-MS analysis of the *n*-hexane extract of *P. artemisioides* roots showed the presence of 25 compounds (Figure 1, Table S1, Supporting Information). In some cases, the MS/MS spectra allowed some compounds to be assigned as belonging to a specific class of specialized metabolites. In particular, the MS/MS spectra of compounds **3**, **5**, **6**, **15**, and **18** showed the loss of H_2_O [M + H-18]^+^ due to the enolic rearrangement of a carbonyl group, and the additional loss of CO from this fragment ion gave the ion [M + H-46]^+^, in agreement with the literature data for tanshinone derivatives.^10^ Peaks corresponding to compounds **7**, **8**, **12**, **14**, **17**, **19**, and **22**–**24** showed a fragmentation pattern typical of abietane derivatives as previously reported in *P. artemisioides* aerial parts.^2^ However, the molecular formula of compounds **2**, **9**, **10**, **11**, **16**, and **20** could not be attributed to diterpenoids previously reported in the literature. With the aim of defining the structures of unknown compounds and to confirm the other identified compounds unambiguously, the isolation of compounds and their structure elucidation by NMR experiments were carried out.

**Figure 2:**
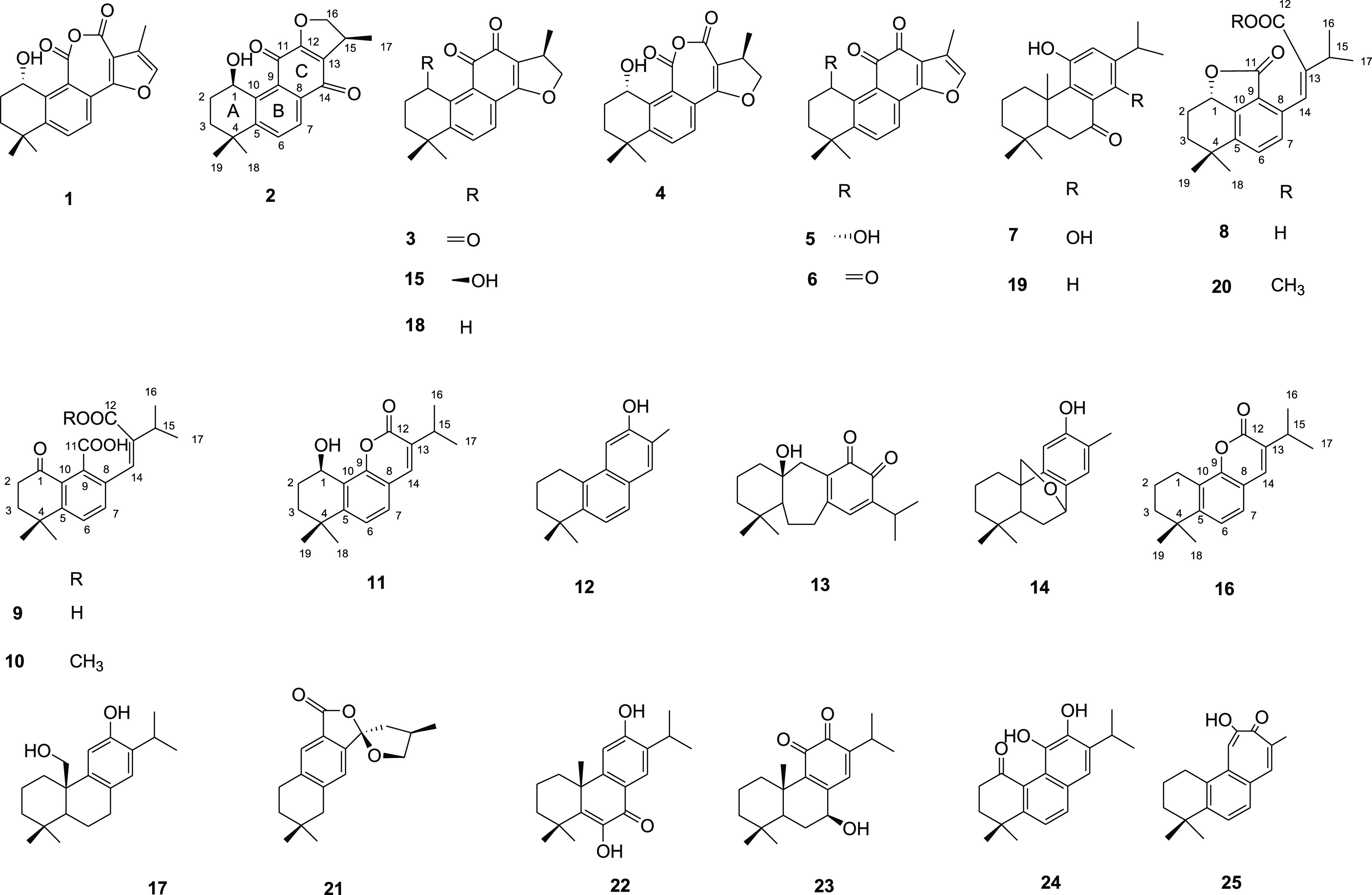
Compounds isolated from *P. artemisioides* roots.

The *n*-hexane extract of *P. artemisioides* roots was fractionated by column chromatography on silica gel, and the fractions were purified by semipreparative HPLC-RI and HPLC-UV, leading to the isolation and identification of six new diterpenoids (**2**, **9**–**11**, **16**, and **20**) along with 19 known diterpenoids (**1**, **3**–**8**, **12**–**15**, **17**–**19**, **21**–**25**) (Figure 2). The structures of the isolated compounds were established by 1D and 2D NMR spectroscopy in combination with mass spectrometry.

The LC-ESI/LTQOrbitrap/MS spectrum of compound **2** (*m*/*z* 313.1425 [M + H]^+^, calcd for C_19_H_21_O_4_, 313.1434) and the ^13^C NMR data supported a molecular formula of C_19_H_20_O_4_. The ^1^H NMR spectrum showed signals for two tertiary methyl groups at δ 1.31 (s) and δ 1.48 (s), a secondary methyl group at δ 1.40 (d, *J* = 7.5 Hz), an oxygenated methylene group at δ 4.49 (dd, *J* = 4.5, 12.2 Hz) and 4.75 (dd, *J* = 4.5, 12.2 Hz), a secondary alcoholic function at δ 5.43 (t, *J* = 3.5, Hz), as well as two signals related to *ortho*-coupled aromatic protons at δ 7.65 and 8.10 (each, d, *J* = 8.5 Hz). The ^13^C NMR spectrum of **2** showed 19 carbon signals (Table 1). Analysis of the carbon resonances revealed the presence of three methyl carbons (δ 16.6, 30.7, and 31.2), two sp^3^ methylene carbons (δ 28.4 and 33.1), one sp^3^ methine carbon (δ 27.6), one sp^3^ quaternary carbon (δ 35.8), one oxygenated methine (δ 61.3), one oxygenated methylene carbon (δ 74.4), two aromatic methines (δ 125.4 and 125.8), five aromatic quaternary carbons (δ 121.8, 130.7, 132.7, 144.4, and 155.2), and three oxygenated aromatic quaternary carbons (δ 160.6, 178.7, and 182.9). These data suggested the presence of a cryptotanshinone derivative.^11^ The secondary hydroxy group at δ 5.43 was assigned to C-1 based on the COSY correlations showing the presence of a −CH(OH)–CH_2_CH_2_– spin system and was confirmed by the chemical shift of C-4 (δ 35.8). This was recognizable by being the only aliphatic quaternary resonance occurring in the spectrum, and was superimposable with that of C-4 in cryptotanshinone (**18**) (δ 35.8) and thus incompatible with the presence of a hydroxy group at C-3. The chemical shifts of the A ring in compound **2** were comparable with those reported for 1β-hydroxycryptotanshinone (**15**).^12^ The configuration at C-1 was deduced from the ^1^H NMR splitting pattern by comparing with literature data for known related compounds. By comparison with literature data, the hydroxy group at C-1 in **2** was determined to be β-oriented based on the ^1^H NMR splitting pattern of H-1, which appeared as a triplet with a *J* value = 3.5 Hz,^1,12^ while in the case of α-OH orientation is reported as a classic ABX system (dd, *J* = 10.0, 5.0 Hz).^13^

**Table 1:** NMR Spectroscopic Data (600 MHz, CD_3_OD) of Compounds **2**, **9**, **10**, and **20**

	**2**		**9**		**10**		**20**	
position	δ_C_, type	δ_H_ (*J* in Hz)	δ_C_, type	δ_H_ (*J* in Hz)	δ_C_, type	δ_H_ (*J* in Hz)	δ_C_, type	δ_H_ (*J* in Hz)
1	61.3, CH	5.43 t (3.5)	200.2	–	201.7, C	–	79.7, CH	5.33 dd (5.8, 12.3)
2	28.4, CH_2_	2.06 m, 2.03 m	36.4, CH_2_	2.74 m	36.4, CH_2_	2.74 m	27.3, CH_2_	1.64, 2.39 m
3	33.1, CH_2_	1.65 dt (2.8), 2.20 m	37.8, CH_2_	2.05 d (8.5)	37.3, CH_2_	2.06 d (8.5)	38.1, CH_2_	1.98 m
4	35.8, C	–	35.2, C	–	35.0, C	–	34.5, C	–
5	155.2, C	–	153.3, C	–	153.8, C	–	143.8, C	
6	125.8, C	7.65 d (8.5)	126.5, CH	7.56 d (8.5)	124.8, CH	7.38 d (8.5)	131.8, CH	7.55 d (8.5)
7	125.4, C	8.10 d (8.5)	135.0, CH	7.44 d (8.5)	133.4, CH	7.38 d (8.5)	130.0, CH	7.31 d (8.5)
8	121.8, C	–	127.8, C	–	131.9, C	–	135.1, C	–
9	144.4, C	–	139.2, C	–	139.9	–	121.3, C	–
10	132.7, C	–	142.5, C	–	142.4, C	–	149.2, C	–
11	182.9, C	–	173.0, C		173.0, C		171.9, C	–
12	160.6, C	–	176.2, C		172.3, C		172.2, C	–
13	130.7, C	–	139.7, C	–	142.8, C		145.0, C	–
14	178.7, C	–	121.6, CH	6.38 d (1.2)	128.7, CH	6.89 d (1.2)	125.4, CH	7.24 s
15	27.6, CH	3.33 overlapped	34.0, CH	2.75 m	34.5, CH	2.76 m	35.6, CH	2.84 sept (6.8)
16	74.4, CH_2_	4.49 dd (4.5, 12.2), 4.75 dd (4.5, 12.2)	21.7, CH_3_	1.20 d (7.5)	21.6, CH_3_	1.19 d (7.5)	21.7, CH_3_	1.23 d (6.8)
17	16.6, CH_3_	1.40 d (7.5)	21.7, CH_3_	1.20 d (7.5)	21.6, CH_3_	1.19 d (7.5)	21.7, CH_3_	1.23 d (6.8)
18	31.2, CH_3_	1.48 s	30.0, CH_3_	1.42 s	29.9, CH_3_	1.41 s	31.0, CH_3_	1.48 s
19	30.7, CH_3_	1.31 s	30.0, CH_3_	1.42 s	29.9, CH_3_	1.41 s	31.8, CH_3_	1.22 s
OCH_3_-12	–	–	–	–	51.5, CH_3_	3.60 s	52.2, CH_3_	3.66 s

Notably, some differences were evident both in the chemical shifts and in the 2D-NMR correlations of the dihydrofuran ring in **2** when compared to corresponding data in cryptotanshinone (**15**). A comparison of the ^1^H NMR spectra of compounds **2** and **15** revealed that H-7, which resonated at δ 7.55 in **15**, is shifted downfield to δ 8.10 in **2**, as reported for isocryptotanshinone.^14^ Moreover, key HMBC correlations between the proton signals at δ 8.10 (H-7) and δ 3.33 (H-15) with the carbon resonance at δ 178.7 (C-14) were observed. The presence of an isocryptotanshinone derivative was also confirmed by the absence of the HMBC correlations between H_2_-16 with the carbon resonance at C-14, observed for the cryptotanshinone derivative (**15**). Thus, the structure of **2** was assigned as 1β-hydroxyisocryptotanshinone.

The LC-ESI/LTQOrbitrap/MS spectrum of compound **9** (*m*/*z* 353.1349 [M + Na]^+^, calcd for C_19_H_22_O_5_Na, 353.1359) and the ^13^C NMR data supported a molecular formula, C_19_H_22_O_5_. The ^1^H NMR spectrum showed signals for two tertiary methyl groups at δ 1.42 (6 H, s), two secondary methyl groups at δ 1.20 (6 H, d, *J* = 7.5 Hz), two signals related to *ortho*-coupled aromatic protons at δ 7.56 and 7.44 (each, d, *J* = 8.5 Hz), and an olefinic proton at δ 6.38 (d, *J* = 1.2 Hz). Analysis of the 19 carbon resonances in the ^13^C NMR spectrum (Table 1) of **9** revealed the presence of four methyl carbons (δ 30.0 and 21.7, each 2C), two sp^3^ methylene carbons (δ 36.4 and 37.8), one sp^3^ methine carbon (δ 34.0), one sp^3^ quaternary carbon (δ 35.2), one carbonyl carbon (δ 200.2), two aromatic methines (δ 126.5 and 135.0), five aromatic quaternary carbons (δ 127.8, 139.2, 139.7, 142.5, and 153.3), and two carboxylic acid groups (δ 173.0, 176.2). On the basis of the NMR data, the presence of an abietane derivative was deduced. Further structural analysis of **9** through the COSY, HSQC, and HMBC spectra confirmed the presence of a tetrahydronaphthalene ring (C-1–C-10) with a carbonyl function at C-1 (δ 200.2) and a geminal dimethyl group at C-4. Moreover, the isopropyl group was assigned through the HMBC correlations between the proton H-15 with the carbon resonances C-12, C-13, and C-14. The detailed analysis of the C ring showed two free carboxylic acid functionalities (δ 173.0, 176.2), which was in agreement with the accurate molecular mass obtained by HRMS, suggesting the cleavage between carbons 11 and 12. The geometry of the olefin (C-13–C-14) was deduced to be *Z* by a ROESY correlation for H-14/H-15. Thus, the structure of compound **9** was elucidated as shown in Figure 2 and named perovskin A.

The LC-ESI/LTQOrbitrap/MS spectrum of compound **10** (*m*/*z* 367.1503 [M + Na]^+^, calcd for C_20_H_24_O_5_Na, 367.1510) and its ^13^C NMR data supported a molecular formula of C_20_H_24_O_5_. The analysis of the HRESIMS data suggested a difference of 14 amu from compound **9**, ascribable to a methyl function. Moreover, the NMR data of compound **10** were superimposable on those of **9** except for the presence of a methoxy group (δ_H_ 3.60, δ_C_ 51.5). The HMBC correlation between the proton signal at δ 3.60 with the carbon resonance at δ 172.3 allowed the methoxy group to be positioned at C-12. Therefore, compound **10** was characterized as shown in Figure 2 and named perovskin B.

A detailed analysis of the NMR data of compounds **11** and **16** showed these compounds to possess a similar structure, differing only in one hydroxy group, as confirmed by HRESIMS analysis. The LC-ESI/LTQOrbitrap/MS spectrum of compound **11** (*m*/*z* 309.1453 [M + Na]^+^, calcd for C_18_H_22_O_3_Na, 309.1456) and the ^13^C NMR data supported a molecular formula of C_18_H_22_O_3_. The LC-ESI/LTQOrbitrap/MS spectrum of compound **16** (*m*/*z* 271.1170 [M + H]^+^, calcd for C_18_H_23_O_2_, 271.1693) and the ^13^C NMR data supported the molecular formula, C_18_H_22_O_2_.

The ^1^H NMR spectrum of compound **11** exhibited signals for two tertiary methyl groups at δ 1.28 and 1.44 (each, 3H, s), two secondary methyl groups at δ 1.30 (6H, d, *J* = 6.8 Hz), a secondary alcoholic group a δ 5.31 (t, *J* = 3.5, Hz), two signals related to *ortho*-coupled aromatic protons at δ 7.43 and 7.55 (each, d, *J* = 8.5 Hz), and one aromatic proton at δ 7.74 (s). The ^13^C NMR spectrum of **11** showed 18 carbon signals (Table 2), ascribable to methyl carbons (δ 21.6, 21.6, 31.0 and 31.5), two sp^3^ methylene carbons (δ 28.5 and 33.4), one sp^3^ methine carbon (δ 30.3), one sp^3^ quaternary carbon (δ 35.4), one oxygenated methine (δ 61.6), three aromatic methines (δ 124.0, 128.2 and 138.4), four aromatic quaternary carbons (δ 118.2, 138.8, 133.5, and 151.8), one oxygenated aromatic quaternary carbon (δ 152.5), and one carboxylic acid (δ 162.2). These data suggested, except for the absence of a carbon resonance, the presence of a miltirone derivative.^15^ Analysis of the 2D NMR spectra suggested a difference in the C ring with respect to miltirone. Key HMBC correlations between the proton signals at δ 7.55 (H-7) and 7.74 (H-14) with the carbon resonance at δ 152.5 indicated the presence of an oxygenated aromatic carbon at C-9. A further HMBC correlation between the proton signal at δ 7.74 (H-14) with the carbon resonance at δ 162.2 allowed the carboxylic acid group to be located at C-12. The downfield shift of the carboxylic acid group, along with the oxygenation at C-9, revealed the occurrence of a lactone function. The secondary alcohol group at δ 5.31 was assigned to C-1 based on the COSY correlations showing the presence of a −CH(OH)–CH_2_CH_2_– spin system in the A ring. The hydroxy group at C-1 in **11** was determined to be β-oriented based on the ^1^H NMR splitting pattern of H-1, which appeared as a triplet with a *J* value = 3.5 Hz.^1,12^ Based on these observations, compound **11** was structurally established, and was named perovskin C.

**Table 2:** NMR Spectroscopic Data (600 MHz, CD_3_OD) of Compounds **11** and **16**

	**11**		**16**	
position	δ_C_, type	δ_H_ (*J* in Hz)	δ_C_, type	δ_H_ (*J* in Hz)
1	61.6, CH	5.31 t (3.5)	24.3, CH_2_	2.92 t (6.5)
2	28.5, CH_2_	2.04 m, 2.01 m	19.6, CH_2_	1.92 m
3	33.4, CH_2_	1.60 dt (3.6, 12.5), 2.19 m	39.7, CH_2_	1.76 m
4	35.4, C	–	35.3, C	–
5	151.8, C	–	150.9, C	–
6	124.0, CH	7.43 d (8.5)	123.9, CH	7.39 d (8.5)
7	128.2, CH	7.55 d (8.5)	125.9, CH	7.41 d (8.5)
8	118.2, C	–	117.9, C	–
9	152.5, C	–	151.9, C	–
10	138.8, C	–	124.8, C	–
11	–		–	
12	162.2, C	–	163.9, C	–
13	133.5, C	–	134.7, C	–
14	138.4, CH	7.74 s	138.7, CH	7.72 s
15	30.3, CH	3.08 sept (6.8)	29.9, CH	3.07 sept (6.8)
16	21.6, CH_3_	1.30 d (6.8)	21.7 CH_3_	1.30 d (6.8)
17	21.6, CH_3_	1.30 d (6.8)	21.7, CH_3_	1.30 d (6.8)
18	31.0, CH_3_	1.44 s	31.8, CH_3_	1.36 s
19	31.5, CH_3_	1.28 s	31.8, CH_3_	1.36 s

The comparison of the NMR experiments of compounds **11** and **16** revealed that the latter compound contained no hydroxy group at C-1, as confirmed by the COSY correlations showing the presence of a −CH_2_–CH_2_CH_2_– spin system in the A ring. Therefore, the structure of compound **16** was elucidated as shown in Figure 2 and named perovskin D.

The LC-ESI/LTQOrbitrap/MS spectrum of compound **20** (*m*/*z* 351.1556 [M + Na]^+^, calcd for C_20_H_24_O_4_Na, 351.1561) and the ^13^C NMR data supported a molecular formula of C_20_H_24_O_4_. The ^1^H NMR spectrum exhibited signals for two tertiary methyl groups at δ 1.48 and 1.22, two secondary methyl groups at δ 1.23 (6H, d, *J* = 6.8 Hz), a methoxy group at δ 3.66 (3H, s), a secondary hydroxy group at δ 5.33 (dd, *J* = 5.8, 12.3 Hz), two signals related to *ortho*-coupled aromatic protons at δ 7.55 and 7.31 (each, d, *J* = 8.5 Hz), and one aromatic proton at δ 7.24 (s). The presence of a tetrahydronaphthalene ring (C-1–C-10) with a geminal dimethyl group at C-4 was inferred from the COSY and HMBC spectra. HMBC correlations between the H-15 signal with the carbon resonances C-12, C-13, and C-14 suggested the position of the isopropyl group at C-13. The secondary hydroxy group at δ 5.33 was assigned to C-1 based on the COSY correlations showing the presence of a −CH(OH)–CH_2_CH_2_– spin system in the A ring. Analysis of the ^13^C NMR (Table 1) and 2D NMR spectra suggested that compound **20** could be a derivative of miltiorin D (**8**), also isolated in this investigation, a 11,12-secoabietane diterpene with a γ-lactone ring.^16^ This was confirmed by a downfield shift observed for C-1 (δ 79.7), suggesting that C-1 is connected to C-11, forming a γ-lactone ring, and by the HMBC correlation between the signal at δ 5.33 (H-1) and the carbon resonance at δ 171.9 (C-11). Lactonization between C-1 and C-11 was in agreement with the molecular formula of **20**. The configuration at C-1, in agreement with that reported for miltiorin D, was deduced from the ^1^H NMR splitting pattern (dd, *J* = 12.3, 5.8 Hz), by comparison with literature data for known related compounds possessing a α-hydroxy group at C-1.^13^ As for miltiorin D, the geometry of the olefin (C-13–C-14) was deduced to be *Z* by a ROESY correlation for H-14/H-15. The methoxy group was assigned to C-12 based on the correlation between the protons at δ 3.66 with the carbon resonance at δ 172.2 (C-12). Based on these observations, compound **20** was assigned as 12-*O*-methylmiltiorin D.

The remaining isolated compounds were identified by spectroscopic data analysis in comparison to values reported in the literature as castanol A (**1**),^13^ 1-oxocryptotanshinone (**3**),^12^ 15-hydroxyanhydride-16*R*-cryptotanshinone (**4**),^4^ 1α-hydroxytanshinone (**5**),^4^ 1-oxotanshinone IIA (**6**),^4^ 1,14-dihydroxy-8,11,13-abietatrien-7-one (**7**),^17^ miltiorin D (**8**),^16^ 12-hydroxy-16,17-bis-nor-simonellite (**12**),^18^ demethylsalvican-11,12-dione (**13**),^19^ przewalskin (**14**),^2^ 1β-hydroxycryptotanshinone (**15**),^12^ pisiferol (**17**),^20^ cryptotanshinone (**18**),^12^ 11-hydroxyabieta-8,11,13-trien-7-one (**19**),^21^ epicryptoacetalide (**21**),^22^ montbretrol (**22**),^23^ 6-deoxysalviphlomone (**23**),^24^ miltiodiol (**24**),^15^ and salviolone (**25**).^4^

The phytochemical investigation of the roots of *P. artemisioides* has led to the isolation of natural abietane-type diterpenoids characterized by an aromatic ring C with different functional groups. They are also named aromatic abietanes and are biosynthesized by two different pathways, the mevalonic acid pathway or the deoxyxylulose phosphate pathway, involving a sequential pair of cyclization and/or rearrangement reactions of geranylgeranyl diphosphate.^25^ Compounds **7**, **14**, **17**, **19**, **22**, and **23** belong to this group. In addition to the abietanes possessing an aromatic C-ring, several co-occurring metabolites were found, such as quinonoid tanshinones characterized by a phenanthrenequinone core, including the *ortho*-quinones **3**, **5**, **6**, **15**, and **18** and the *para*-quinone **2**. From the roots of *P. artemisioides,* compounds with a rearranged C ring (**1**, **4**, **8**–**11**, **16**, and **20**), probably deriving from the quinonoid tanshinones, were also isolated. Compounds **1** and **4** are an example of 20-norabietane diterpenoids with a seven-membered-ring anhydride as the C ring. It has been reported that a possible mechanism for their formation is a photo-oxidation of tanshinones.^26^ Compounds **8**–**10** and **20** could derive from the 20-norabietane diterpenes with an *ortho*-quinone C-ring through a C-11/C-12 oxidative cleavage. For compounds **9** and **10**, herein reported for the first time, the presence of a carbonyl function at C-1 would prevent the formation of a γ-lactone ring between C-1 and C-11, as apparent in compounds **8** (miltiorin D) and **20**, which are both characterized by hydroxylation at C-1. This biosynthetic pathway could determine the formation of the new compounds **11** and **16**, where the C-11/C-12 cleavage may be followed by the loss of the carboxylic acid group at C-11, hydroxylation at C-9 and the formation of an ester linkage between the hydroxy group at C-9 and the carboxyl group at C-12. Along with the new compounds, characterized substances belonging to the class of 20-norabietane diterpenoids with a seven-membered-ring anhydride as the C ring (**1**, **4**) or derived from an *ortho*-quinone C-ring through a C-11/C-12 oxidative cleavage (**8**), are reported herein for the first time from the genus *Perovskia*.

### Effects of the Isolated Compounds on Macrophage Cell Viability and NO Release

The *n*-hexane extract of *P. artemisioides* and selected isolated compounds were evaluated for their effects on the viability of macrophages. After 24 h of treatment with the plant extract (100–12.5 μg/mL) or compounds (100–12.5 μM), the antiproliferative activity of the J774A.1 cells was evaluated using a MTT assay. The extract exerted the inhibition of J774A.1 cell proliferation activity (87.99 ± 0.42%, 42.76 ± 0.33%, and 6.37 ± 0.42% respectively, at 100, 50, and 25 μg/mL), while compounds **16**–**18** and **20**–**25** exerted significant effects on J774A.1 cell proliferation activity only at a concentration of 100 μM.

**Figure 3:**
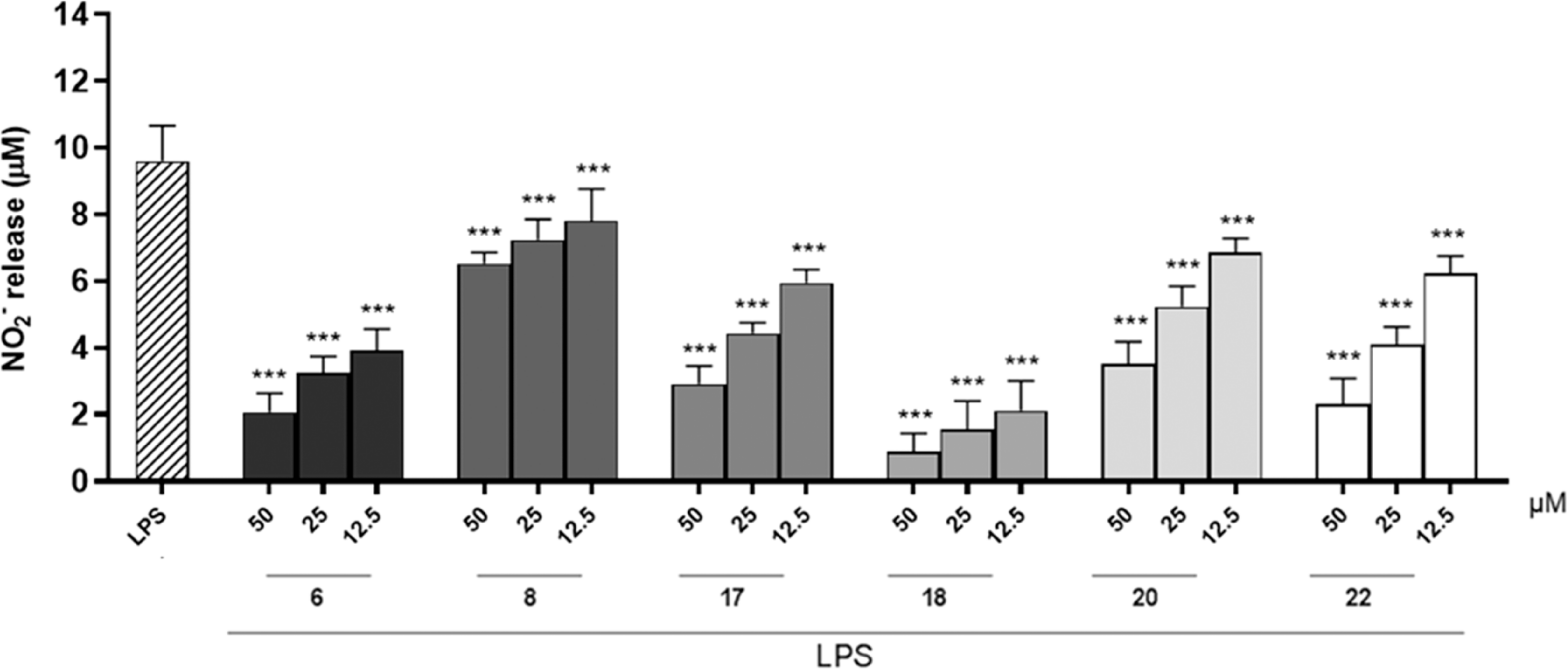
Effect of test compounds (50–12.5 μM) on NO release by J774A.1 macrophages stimulated with lipopolysaccharide from *E. coli* (LPS; 10 μg/mL) and detected by a Griess assay. Results are shown as means ± SEM of NO_2_^–^ release vs LPS alone. *** Denotes *p* < 0.001 vs LPS.

Substantial evidence has indicated that chronic inflammation can modulate different pathological stages.^27^ Along with cytokines and chemokines released during inflammation, macrophages and infiltrating neutrophils release nitric oxide (NO), a highly reactive, short-lived free radical known both as an important mediator in various biological functions and, when produced in large quantities, as a modulator of the inflammatory response through a variety of different pathways.^28^ NO synthesis results from the conversion of the amino acid l-arginine to l-citrulline, mediated by a family of enzymatic isoforms known as nitric oxide synthases (NOS). During an inflammatory response, inducible nitric oxide synthase (iNOS) is the isoform responsible for NO production, induced by both pro-inflammatory cytokines such as interleukin (IL)-1, IL-12, interferon (INF)-γ, or tumor necrosis factor (TNF)-α, and by the lipopolysaccharide of *Escherichia coli*, a constituent of the Gram-negative bacterial wall.^29,30^ The cytotoxic effects of NO target DNA, proteins, or lipids, but also numerous proteins and enzymes critical for cell survival and signaling, including molecules involved in cytokine signaling such as JAK or STAT proteins, the NF-κB/IκB-dependent pathway or MAPKs, some G proteins, transcription factors or hormones controlling the inflammatory process at the central level.^30,31^ Therefore, in the present work, NO generation was measured as nitrite (NO_2_^–^) detected by the Griess reagent and expressed as μM concentrations calculated using a NO_2_^–^ sodium standard curve. The results obtained indicated that among the compounds tested (50–12.5 μM), **6** showed the greatest potential for reducing NO_2_^–^. Specifically, compounds **6**, **8**, **17**, **18**, **20**, and **22** inhibited NO release at all tested concentrations (*p* < 0.001 vs LPS; Figure 3). L-NAME (1 μM), a known NO inhibitor, was used as a reference substance and inhibited NO release by 51.18 ± 0.67%. Thus, considering their marked activity observed on NO release, subsequent studies were performed evaluating only the above compounds.

### Effect of Compounds **6**, **8**, **17**, **18**, **20**, and **22** on iNOS Release

**Figure 4:**
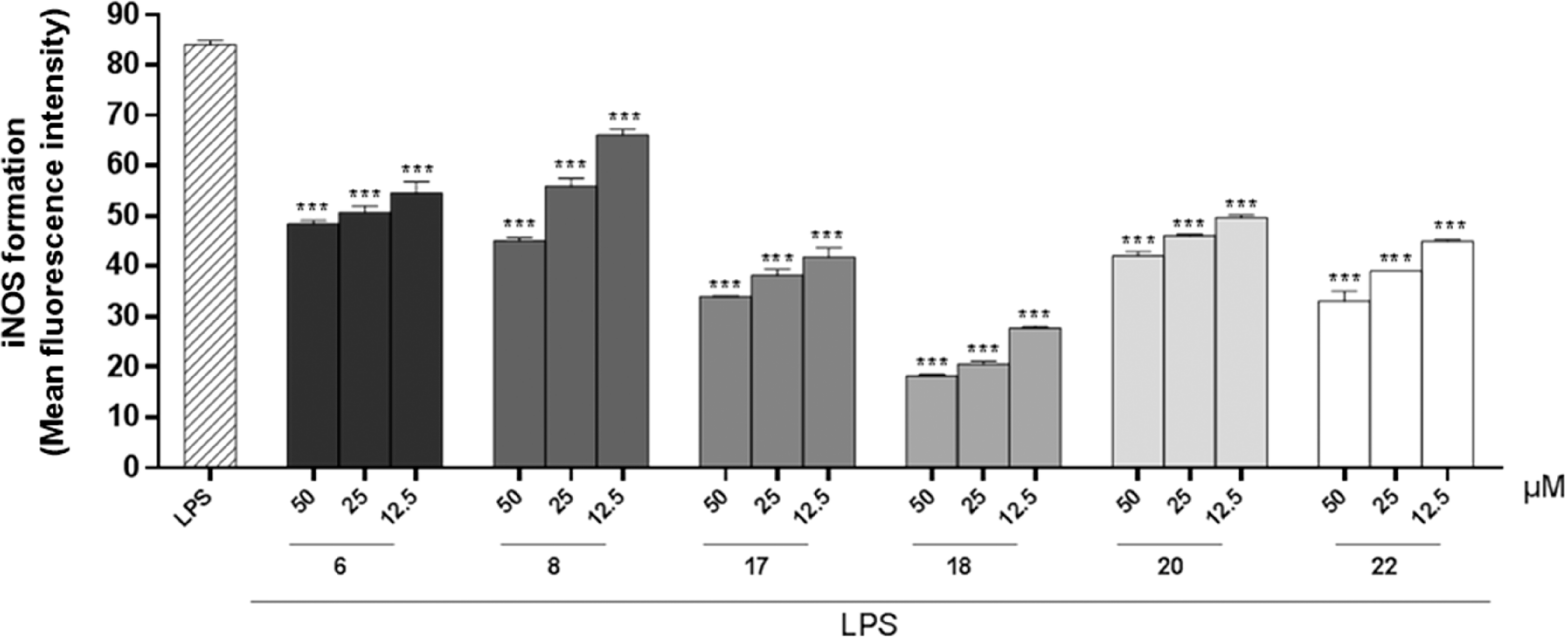
Effect of test compounds (50–12.5 μM) on iNOS formation in J774A.1 macrophages stimulated with lipopolysaccharide from *E. coli* (LPS; 10 μg/mL) and detected by cytofluorimetry. Results are shown as means ± SEM of the mean fluorescence intensity of iNOS formation vs LPS alone. *** Denotes *p* < 0.001 vs LPS.

Under inflammatory conditions, the iNOS enzyme is overexpressed, and therefore the effects of compounds on iNOS formation in macrophages were evaluated. J774A.1 cells were treated with compounds **6**, **8**, **17**, **18**, **20**, and **22** (50–12.5 μM) for 1 h and then simultaneously with LPS (10 μg/mL) for 24 h, as described previously.^32^ All compounds tested inhibited iNOS expression during the inflammatory conditions used at all test concentrations (50–12.5 μM; *p* < 0.001 vs LPS; Figure 4). L-NAME was used as a reference substance and inhibited iNOS formation by 47.06 ± 0.52%.

### Effect of Compounds **6**, **8**, **17**, **18**, **20**, and **22** on COX-2 Formation

**Figure 5:**
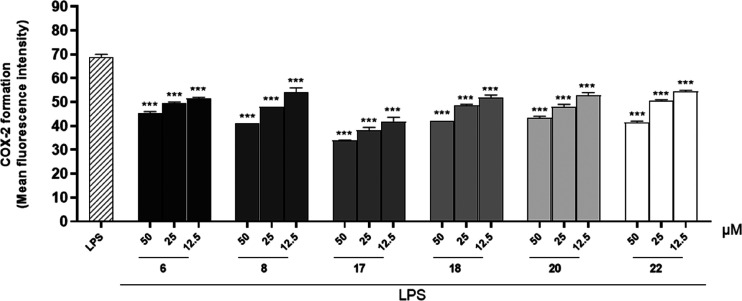
Effect of test compounds (50–12.5 μM) on COX-2 formation in J774A.1 macrophages stimulated with lipopolysaccharide from *E. coli* (LPS; 10 μg/mL) and detected by cytofluorimetry. Results are shown as means ± SEM of mean fluorescence intensity of COX-2 formation vs LPS alone. *** Denotes *p* < 0.001 vs LPS.

Mediators that play a critical role during the inflammatory process may also interact with each other and amplify the pro-inflammatory response. NO directly modulates the activity of the enzyme cyclooxygenase type 2 (COX-2), promoting an increase of prostaglandin production. COX-2 is present constitutively in the kidney, central nervous system, and intestine, but, in being also an inducible isoform, it can be regulated and stimulated by specific events involved in inflammation, enabling this enzyme responsible for the biosynthesis of prostanoids.^33^ Therefore, the anti-inflammatory potential of the compounds was also assessed through their effects on COX-2 formation in macrophages under inflammatory conditions. J774A.1 cells were treated with compounds **6**, **8**, **17**, **18**, **20**, and **22** (50–12.5 μM) for 1 h and then simultaneously with LPS (10 μg/mL) for 24 h, as described previously.^32^ The results obtained indicated that all compounds significantly inhibited COX-2 formation during inflammatory conditions at all tested concentrations (50–12.5 μM; *p* < 0.001 vs LPS; Figure 5). Indomethacin, used as a reference drug, inhibited COX-2 formation by 47.82 ± 0.33%.

### Effect of Compounds **6** and **18** on Nitrotyrosine Formation and ROS Release

Chronic inflammation may also be associated with increased levels of reactive oxygen species (ROS) and nitrogen species (RNS), which can induce oxidative stress, another key component in the pathogenesis of the inflammatory process. Oxidative stress occurs when antioxidant defense systems lose their ability to eliminate excess free radicals or reactive intermediates. This condition causes further damage to DNA, proteins, and lipids and further activation of pro-inflammatory pathways.^34^ In this scenario, the interaction between high levels of NO and molecular oxygen (O_2_)-derived reactive oxygen species causes post-translational oxidative modification of proteins. Tyrosine residues are one of the main targets of oxidant molecules that undergo chemical modifications affecting the chemical and physical properties of proteins. In particular, the reaction between NO and the superoxide radical (O^2–^) leads to the formation of peroxynitrite (ONOO^–^), a highly reactive nitrating agent, which, in turn, leads to the formation of 3-nitrotyrosine through a *S*-nitrosylation reaction. Under physiological conditions, small amounts of nitrotyrosine are produced, but under inflammatory conditions, the levels are elevated significantly. Therefore, nitrotyrosine is an important marker of nitro-oxidative stress.^35^ Thus, since compounds **6** and **18** showed the most potent activities in reducing NO release, the effect of these compounds on nitrotyrosine formation in J774A.1 cells during inflammatory conditions was evaluated.

**Figure 6:**
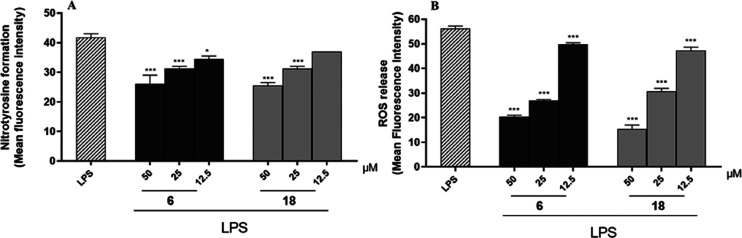
Effect of compounds **6** and **18** (50–12.5 μM) on nitrotyrosine formation (A) and ROS release (B) in J774A.1 macrophages stimulated with lipopolysaccharide from *E. coli* (LPS; 10 μg/mL) and detected by cytofluorimetry. Results are shown as means ± SEM of mean fluorescence intensity of nitrotyrosine (A) and ROS release (B) formation vs LPS alone. *** and * denote, respectively, *p* < 0.001 and *p* < 0.05 vs LPS.

Macrophages were treated with compounds **6** and **18** (50–12.5 μM) for 1 h and then simultaneously with LPS (10 μg/mL) for 24 h, as described previously.^32^ The results obtained indicated that compound **6** inhibited nitrotyrosine formation at all concentrations tested (50–12.5 μM; *p* < 0.05 vs LPS; Figure 6A), whereas compound **18** inhibited nitrotyrosine formation only at the higher concentrations used (50–25 μM; *p* < 0.001 vs LPS; Figure 6A).

Subsequently, to confirm the antioxidant potential of compounds **6** and **18**, their effects on ROS release were also evaluated. ROS includes all oxygen-free radicals containing at least one unpaired electron. Under physiological conditions, small amounts of ROS are formed that are involved in both cellular processes and act as signaling molecules. In addition, a balance is established between the generation and removal of free radicals. However, the excessive formation of ROS contributes to oxidative stress and causes damage at the molecular and cellular levels, also contributing to the development of numerous free radical-mediated pathologies.^36^ Thus, J774A.1 cells were treated with compounds **6** and **18** (50–12.5 μM) for 1 h and then simultaneously with LPS (10 μg/mL) for 24 h, as described previously.^37^ The results obtained indicated that both compounds inhibited ROS release at all concentrations tested (12.5–50 μM; *p* < 0.001 vs LPS; Figure 6B).

## Experimental Section

### General Experimental Procedures

Optical rotations were obtained on a JASCO 2000 polarimeter. IR measurements were carried out on a Bruker IFS-48 spectrometer. NMR experiments were acquired on a Bruker Ascend-600 NMR spectrometer (Bruker BioSpin GmBH, Rheinstetten, Germany) equipped with a Bruker 5 mm PATXI probe. DQF-COSY, HSQC, HMBC, and ROESY spectra were acquired in methanol-*d*_4_ (99.95%, Sigma-Aldrich), and standard pulse sequences and phase cycling were used. The 1D and 2D NMR data were processed by TOPSPIN 3.2 software. For LC-MS a Thermo Scientific liquid chromatography system having a quaternary Accela 600 pump, an Accela autosampler, connected to a linear Trap-Orbitrap hybrid mass spectrometer (LTQ-Orbitrap XL, Thermo Fisher Scientific, Bremen, Germany) with electrospray ionization (ESI), operating in positive-ion mode, was used. Calibration of the LTQ-Orbitrap system was performed according to the manufacturer’s instructions using a mixture of caffeine, methionine-arginine-phenylalanine-alanine-acetate (MRFA), sodium dodecyl sulfate, sodium taurocholate, and Ultramark 1621. Data were collected and analyzed using the software provided by the manufacturer. Xcalibur software version 2.1 was used for the instrument control, data acquisition, and data analysis. Silica gel was used for chromatography. HPLC separations were carried out by two HPLC instruments: a Waters 590 system equipped with a Waters R401 refractive index detector and an Agilent 1260 Infinity system (Agilent Technologies, Palo Alto, CA, USA), equipped with a binary pump (G-1312C), and a UV detector (G-1314B).

### Plant Material

The roots of *P. artemisioides* were collected in July 2017 at the Taftan Mt. Dareh gol region, Sistan and Baluchestan Province, Iran (GPS coordinates 28°36′28.94″N; 61°4′40.40″E). The whole parts of the species were identified by the botanist Dr. A. Sonboli, and a voucher specimen of the whole plant (MPH-2725) was deposited at the herbarium of Medicinal Plants and Drugs Research Institute, Shahid Beheshti University, Tehran, Iran.

### LC-HRMS/MS Analysis

Qualitative LC-HRMS/MS analysis was performed using a system of liquid chromatography coupled to the hybrid mass spectrometer LTQ-Orbitrap XL, which combines a linear trap quadrupole (LTQ) and an Orbitrap mass analyzer.^38^ A C_18_ reversed-phase (RP) column (150 mm × 2.00 mm) Luna C_18_ 5 μm (Waters, Milford, MA) at a flow rate of 0.2 mL/min was selected, with water plus 0.1% formic acid and acetonitrile plus 0.1% formic acid used as phases A and B, respectively. For the experimental conditions and HPLC gradient, the same conditions as previously reported for *Perovskia artemisioides* aerial parts were used.^2^ The autosampler was set to inject 4 μL of *n*-hexane extract (0.5 mg/mL). The mass range was from *m*/*z* 150 to 1600 with a resolution of 30,000. A data-dependent scan experiment was performed for fragmentation, selecting precursor ions corresponding to the first and the second most intense ions from the LC-HRMS spectrum and using a normalization collision energy of 30%, a minimum signal threshold of 250, and an isolation width of 2.0.

### Extraction and Isolation

The dried roots of *P. artemisioides* (930 g) were milled and macerated at room temperature with *n*-hexane (3 × 3 L, 72 h) and then with chloroform (3 × 3 L). The dried *n*-hexane extract (23.26 g) was fractionated by silica gel column chromatography (100 × 4.5 cm, 70–230 mesh) with a step gradient elution of *n*-hexane/EtOAc (100:0 to 0:100, v/v) as mobile phases, to give 303 fractions monitored by TLC. Based on TLC analysis, fractions with similar compositions were combined to yield 32 pooled fractions.

Fraction 19 (187–198) (100.0 mg) was chromatographed by semipreparative HPLC-RI with a Waters XTerra Prep MSC_18_ column (300 × 7.8 mm i.d.), using MeOH–H_2_O (6.1:3.9) as mobile phase (flow rate 2.5 mL/min), to yield compounds **2** (3.8 mg, *t*_R_ = 14.3 min), and **4** (5.9 mg, *t*_R_ = 18.0 min). Fraction 16 (61–66) (270.0 mg) was chromatographed using MeOH–H_2_O (6:4) as mobile phase (flow rate 2.5 mL/min) to afford compound **1** (2.9 mg, *t*_R_ = 6.2 min).

Fractions 4, 5, 7, 8, 10, 13, and 20 were each subjected to a RP-HPLC-UV separation. The elution gradient was obtained using water with 0.1% formic acid as eluent A and acetonitrile with 0.1% formic acid as B at a flow rate of 2.0 mL/min, with the detection wavelength being 280 nm, and the purification steps were performed at room temperature. For fraction 4 (41–65) (100 mg), the HPLC gradient started from 40% B and proceeded from 40 to 80% B in 35 min, from 80 to 90% in 5 min, from 90 to 100% B in 5 min and at 100% B for 10 min, to obtain compounds **12** (1.0 mg, *t*_R_ = 16.78 min), **23** (3.3 mg, *t*_R_ = 20.92 min), and **25** (5.1 mg, *t*_R_ = 22.24 min). For fractions 5, 8, and 10, the same gradient was used as for fraction 4. Fraction 5 (66–84) (100 mg) was purified to yield compound **21** (7.9 mg, *t*_R_ = 16.46 min), fraction 8 (94–110) (100 mg) was subjected to RP-HPLC-UV to obtain compounds **9** (3.8 mg, *t*_R_ = 11.17 min) and **24** (3.5 mg, *t*_R_ = 40.13 min), and fraction 10 (117–119) (100.0 mg) gave compounds **15** (7.9 mg, *t*_R_ = 16.73 min), **11** (7.6 mg, *t*_R_ = 18.80 min), **17** (2.5 mg, *t*_R_ = 29.42 min), and **13** (2.5 mg, *t*_R_ = 32.40 min).

For fraction 7 (94–110) (100 mg) the HPLC gradient started from 35% B for 5 min and proceeded from 40 to 80% B in 35 min, from 80 to 90% in 5 min, from 90 to 100% B in 5 min and at 100% B for 10 min to obtain compounds **10** (2.7 mg, *t*_R_ = 15.36 min), **14** (7.5 mg, *t*_R_ = 20.68 min), **20** (2.8 mg, *t*_R_ = 27.86 min), and **19** (3.3 mg, *t*_R_ = 36.5 min). For fractions 13 and 20 the same gradient was used as for fraction 7. Hence, fraction 13 (133–146) (100 mg) was chromatographed to yield compounds **5** (2.8 mg, *t*_R_ = 23.01 min) and **6** (2.6 mg, *t*_R_ = 15.07 min), while fraction 20 (199–204) (100 mg) was purified to obtain compounds **3** (2.9 mg, *t*_R_ = 13.39 min) and **8** (3.4 mg, *t*_R_ = 27.79 min).

Fraction 1 (1–24) (100.0 mg), using semipreparative UV-HPLC (from 45 to 80% B for 35 min, from 80 to 90% for 5 min, from 90 to 100% B for 5 min and then 100% B for 10 min) was purified to yield compound **16** (6.5 mg, *t*_R_ = 35.93 min). Fraction 3 (36–40) (100 mg) was separated further to give compounds **22** (1.5 mg, *t*_R_ = 25.00 min) and **7** (2.3 mg, *t*_R_ = 34.25 min). Compound **18** was obtained directly from the silica gel column as a major constituent (20.6 mg).

#### 1β-Hydroxyisocryptotanshinone (**2**)

Colorless amorphous solid; [α]^25^_D_ + 4 (*c* 0.05, MeOH); IR (KBr), ν_max_ 3260, 1730, 1466, 1378, 1085 cm^–1^; for the ^1^H NMR (CD_3_OD, 600 MHz) and ^13^C NMR (CD_3_OD, 150 MHz) data, see Table 1; HRESIMS *m*/*z* 313.1425 [M + H]^+^ (calcd for C_19_H_21_O_4_, 313.1434).

#### Perovskin A (**9**)

Colorless amorphous solid; IR (KBr) ν_max_ 3360, 1656, 1432, 1298, 1053 cm^–1^; for the ^1^H NMR (CD_3_OD, 600 MHz) and ^13^C NMR (CD_3_OD, 150 MHz) data, see Table 1; HRESIMS *m*/*z* 353.1349 [M + Na]^+^ (calcd for C_19_H_22_O_5_Na, 353.1359).

#### Perovskin B (**10**)

Colorless amorphous solid; IR (KBr) ν_max_ 3372, 1665, 1422, 1308, 1066 cm^–1^; for the ^1^H NMR (CD_3_OD, 600 MHz) and ^13^C NMR (CD_3_OD, 150 MHz) data, see Table 1; HRESIMS *m*/*z* 367.1503 [M + Na]^+^ (calcd for C_20_H_24_O_5_Na, 367.1510).

#### Perovskin C (**11**)

Colorless amorphous solid; [α]^25^_D_ + 4 (*c* 0.06, MeOH); IR (KBr), IR (KBr) ν_max_ 3385, 1676, 1462, 1383, 1052 cm^–1^; for the ^1^H NMR (CD_3_OD, 600 MHz) and ^13^C NMR (CD_3_OD, 150 MHz) data, see Table 2; HRESIMS *m*/*z* 309.1453 [M + Na]^+^ (calcd for C_18_H_22_O_3_Na, 309.1456).

#### Perovskin D (**16**)

Colorless amorphous solid; IR (KBr) ν_max_ 1656 1459, 1385, 1055 cm^–1^; for the ^1^H NMR (CD_3_OD, 600 MHz) and ^13^C NMR (CD_3_OD, 150 MHz) data, see Table 2; HRESIMS *m*/*z* 271.1170 [M + H]^+^ (calcd for C_18_H_23_O_2_, 271.1693).

#### 12-O-Methylmiltiorin D (**20**)

Colorless amorphous solid; [α]^25^_D_ + 84 (*c* 0.18, MeOH); IR (KBr) ν_max_ 1640, 1455, 1382, 1051 cm^–1^; for the ^1^H NMR (CD_3_OD, 600 MHz) and ^13^C NMR (CD_3_OD, 150 MHz) data, see Table 1; HRESIMS *m*/*z* 351.1556 [M + Na]^+^ (calcd for C_20_H_24_O_4_Na, 351.1561).

### Cell Culture

The murine macrophage cell line (J774A.1) was obtained from the American Tissue Culture Collection (ATCC). J774A.1 cells were routinely maintained in the presence of Dulbecco’s modified Eagle’s medium (DMEM; 4 g/L glucose) containing 10% (v/v) fetal bovine serum (FBS), penicillin (100 μg/mL), and streptomycin (100 μg/mL). Cells were grown at 37 °C in a humidified atmosphere of 5% CO_2_/95% air.

### Cell Viability Test

J774A.1 macrophages were plated (5 × 104 cells/well) on 96-well plates and allowed to adhere for 3 h at 37 °C in 5% CO_2_/95% air. Thereafter, the medium was replaced with fresh medium alone or containing serial dilutions of the extract (100–12.5 μg/mL) or compounds **1**–**25** (100–12.5 μM), and incubation was performed for 24 h. Cell viability was assessed using 3-(4,5-dimethylthiazol-2-yl)-2,5-diphenyl tetrazolium bromide (MTT; 5 mg/mL).^39^ After 24 h, 25 μL of MTT were added, and after another 3 h, 100 μL of dimethyl sulfoxide (DMSO) were added. After 15 min, a microplate spectrophotometer (Titertek Multiskan MCC/340-DASIT, Cornaredo, Milan, Italy), equipped with a 570 nm filter, was used to measure the optical density (OD) in each well. The antiproliferative activity of J774A.1 cells was calculated as the % of dead cells: 100 – [(OD treated/OD control) × 100]. 6-Mercaptopurine (1 μM) was used as the reference compound.

### Measurement of NO Release

J774A.1 cells were cultured and then plated (5 × 104 cells/well) in 96-well microtiter plates and allowed to adhere at 37 °C in 5% CO_2_/95% air, as described previously.^32^ After 3 h, the culture medium was replaced with fresh medium alone or containing serial dilutions of compounds **1**–**3**, **5**–**12**, or **14**–**25** (50–12.5 μM) for 1 h. Lipopolysaccharide of *Escherichia coli* (LPS; 10 μg/mL) was then added as a pro-inflammatory stimulus. After 24 h, NO production was evaluated as nitrite (NO^2–^), the index of NO released by macrophages in the culture medium using Griess reagent and incubated at room temperature for 10 min.^40^ The absorbance was measured at 550 nm in a microplate reader Titertek (Dasit, Cornaredo, Milan, Italy).

### Detection of iNOS and COX-2 Expression and Nitrotyrosine Formation by Cytofluorimetry

J774A.1 cells were cultured and then plated (5 × 10^4^ cells/well) in 96-well microtiter plates and allowed to adhere at 37 °C in 5% CO_2_/95% air, as described previously.^40^ After 3 h, the culture medium was replaced, and the cells were treated with serial dilutions of compounds **6**, **8**, **17**, **18**, **20**, or **22** (50–12.5 μM) added 1 h before and simultaneously to LPS (10 μg/mL), to evaluate iNOS and COX-2 expression. Nitrotyrosine formation was assessed by treating cells with compounds **6** and **18** (50–12.5 μM), and after 1 h, LPS (10 μg/mL) was added, as described previously.^32^ The compound *N*-nitro-l-arginine methyl ester hydrochloride (L-NAME; 1 μM) and indomethacin (1 μM) were used as positive controls. After 24 h, cells were harvested and washed with phosphate-buffered saline (PBS) solution, incubated for 20 min first in fixing solution and then in perm solution for 30 min. Then, primary antinitric oxide inducible synthase (anti-iNOS; BD Transduction Laboratories, Milan, Italy), anticyclooxygenase-2 (anti-COX-2; BD Transduction Laboratories, Milan, Italy), and with rabbit antinitrotyrosine (Millipore, Billerica, MA, USA) antibodies were added to the cells for 30 min. Then, the secondary antibody, in fixing solution, was added to J774A.1 for 30 min, and fluorescence was then evaluated using a fluorescence-activated cells sorting device (FACSscan; Becton Dickinson, Milan, Italy), and processed using Cell Quest software (version 4; Becton Dickinson, Milan, Italy).^32^

### Intracellular ROS Release Measurement

J774A.1 cells were cultured and then plated (5 × 104 cells/well) in 96-well microtiter plates and allowed to adhere at 37 °C in 5% CO_2_/95% air, as described previously.^40^ After 3 h, the culture medium was replaced and cells were treated with serial dilutions of compounds **6** and **18** (50–12.5 μM), added 1 h before and simultaneously to LPS (10 μg/mL). After 24 h, ROS formation was evaluated using the probe 2′,7′-dichlorofluorescein-diacetate (H_2_DCF-DA). In the presence of intracellular ROS, H_2_DCF is rapidly oxidized to the highly fluorescent 2′,7′-dichlorofluorescein (DCF). Thus, J774A.1 cells were collected, washed twice with phosphate buffer saline (PBS), and then incubated in PBS containing H_2_DCF-DA (10 μM) at 37 °C. After 15 min, fluorescence was evaluated using a FACSscan and processed by Cell Quest software, as previously reported.^37^

### Statistical Analysis of Data

Data are reported as means ± mean standard error (SEM) of at least three independent experiments. Each experiment was conducted in triplicate. Analysis of variance and Bonferroni’s test were used for data analysis to perform multiple comparisons. A *p* value less than 0.05 was considered significant.

## Supporting Information Available

Retention times, molecular formulas, and MS/MS values of compounds **1**–**25**; ESI/LTQOrbitrap spectra; ^1^H NMR spectra; ^13^C NMR spectra; HSQC spectra; HMBC spectra; COSY spectra; and ROESY spectra (PDF)
