# Supplementary material for: Anti-inflammatory Activity of Tanshinone-Related Diterpenes from Perovskia artemisioides Roots
Source: J Nat Prod. 2023 Apr 11;86(4):812–21. doi: 10.1021/acs.jnatprod.2c01004 (PMC10152488; doi:10.1021/acs.jnatprod.2c01004)
Supplement: Supplementary file 1 — np2c01004_si_001.pdf [file np2c01004_si_001.pdf]

Anti-inflammatory Activity of Tanshinone-related Diterpenes from *Perovskia artemisioides* Roots

Zahra Sadeghi,[†,‡,§] Antonietta Cerulli,[‡,§] Stefania Marzocco,[‡] Mahdi Moridi Farimani,[†,*] Milena Masullo[‡], and Sonia Piacente[‡,*]

[†]Department of Phytochemistry, Medicinal Plants and Drugs Research Institute, Shahid Beheshti University, Evin, Tehran, Iran

[‡]Dipartimento di Farmacia, Università degli Studi di Salerno, via Giovanni Paolo II n. 132, 84084 Fisciano (SA), Italy

[*]Corresponding authors:

Tel: +98 21 29904043. Fax: +98 21 22431783. E-mail addres: m_moridi@sbu.ac.ir

Tel.: +39 089969763; Fax:+39 089969602. E-mail address: piacente@unisa.it

Supplementary materials

Table S1. Retention Times (R_t), $[M+H]^+$, $[M+Na]^+$, Molecular Formula, Δ ppm and MS/MS Values of Compounds **1-25** of *n*-Hexane Extract of *P. artemisioides* roots by LC-ESI/LTQOrbitrap/MS.

Figure S1. ESI/LTQOrbitrap spectrum of compound **2**, in positive ion mode.

Figure S2. ^{1}H NMR Spectrum (600 MHz, CD_3OD) of compound **2**.

Figure S3. ^{13}C Spectrum (150 MHz, CD_3OD) of compound **2**.

Figure S4. HSQC Spectrum (CD_3OD) of compound **2**.

Figure S5. HMBC Spectrum (CD_3OD) of compound **2**.

Figure S6. COSY Spectrum (CD_3OD) of compound **2**.

Figure S7. ROESY Spectrum (CD_3OD) of compound **2**.

Figure S8. ESI/LTQOrbitrap spectrum of compound **9**, in positive ion mode.

Figure S9.^{1}H NMR Spectrum (600 MHz, CD_3OD) of compound **9**.

Figure S10. ^{13}C Spectrum (150 MHz, CD_3OD) of compound **9**.

Figure S11. HSQC Spectrum (CD_3OD) of compound **9**.

Figure S12. HMBC Spectrum (CD_3OD) of compound **9**.

Figure S13. COSY Spectrum (CD_3OD) of compound **9**.

Figure S14. ROESY Spectrum (CD_3OD) of compound **9**.

Figure S15. ESI/LTQOrbitrap spectrum of compound **10**, in positive ion mode.

Figure S16.^{1}H NMR Spectrum (600 MHz, CD_3OD) of compound **10**.

Figure S17. ^{13}C Spectrum (150 MHz, CD_3OD) of compound **10**.

Figure S18. HSQC Spectrum (CD_3OD) of compound **10**.

Figure S19. HMBC Spectrum (CD_3OD) of compound **10**.

Figure S20. COSY Spectrum (CD_3OD) of compound **10**.

Figure S22. ESI/LTQOrbitrap spectrum of compound **11**, in negative ion mode.

Figure S23. ^{1}H NMR Spectrum (600 MHz, CD_3OD) of compound **11.**

Figure S24. ^{13}C Spectrum (150 MHz, CD_3OD) of compound **11.**

Figure S25. HSQC Spectrum (CD_3OD) of compound **11.**

Figure S26. HMBC Spectrum (CD_3OD) of compound **11.**

Figure S27. COSY Spectrum (CD_3OD) of compound **11.**

Figure S28. ROESY Spectrum (CD_3OD) of compound **11.**

Figure S29. ESI/LTQOrbitrap spectrum of compound **16**, in negative ion mode.

Figure S30. ^{1}H NMR Spectrum (600 MHz, CD_3OD) of compound **16.**

Figure S31. ^{13}C Spectrum (150 MHz, CD_3OD) of compound **16.**

Figure S32. HSQC Spectrum (CD_3OD) of compound **16.**

Figure S33. HMBC Spectrum (CD_3OD) of compound **16.**

Figure S34. COSY Spectrum (CD_3OD) of compound **16.**

Figure S36. ^{1}H NMR Spectrum (600 MHz, CD_3OD) of compound **20.**

Figure S37. ^{1}H NMR Spectrum (600 MHz, CD_3OD) of compound **20.**

Figure S38. ^{13}C Spectrum (150 MHz, CD_3OD) of compound **20.**

Figure S39. HSQC Spectrum (CD_3OD) of compound **20.**

Figure S40. HMBC Spectrum (CD_3OD) of compound **20.**

Figure S41. COSY Spectrum (CD_3OD) of compound **20.**

Table S1. Retention Times (R_t), $[M+H]^+$, $[M+Na]^+$, Molecular Formula, Δ ppm, and MS/MS Values of Compounds **1-25** of *n*-Hexane Extract of *P. artemisioides* roots by LC-ESI/LTQOrbitrap/MS.

	R_t	$[M+H]^+$	Mol Formula	Δppm	MS/MS	Name
**1**	3.52	327.1218	$C_{19}H_{18}O_5$	-2.69	309, 283	castanol A
**2**	5.02	313.1423	$C_{19}H_{20}O_4$	-3.72	269, 294, 252	1β-hydroxy-isocryptotanshinone
**3**	7.73	311.1270	$C_{19}H_{18}O_4$	-2.40	265, 283, 293	1-oxocryptotanshinone
**4**	10.41	329.1374	$C_{19}H_{20}O_5$	-2.92	314, 267	15 hydroxy-anhydride-16R cryptotanshinone
**5**	10.74	311.1269	$C_{19}H_{18}O_4$	-2.59	265, 281, 293	1α-hydroxytanshinone
**6**	12.86	309.1112	$C_{19}H_{16}O_4$	-2.90	281, 263, 235	1-oxotanshinone IIA
**7**	13.25	317.2104	$C_{20}H_{28}O_3$	-2.21	299, 273, 213	1,14-dihydroxy-8,11,13-abietatrien-7-one
**8**	14.73	315.1579	$C_{19}H_{22}O_4$	-3.57	297, 241	miltiorin D
**9**	15.03	331.1534	$C_{19}H_{22}O_5$	-1.96		perovskin A
**10**	15.51	345.1685	$C_{20}H_{24}O_5$	-3.45	327, 313, 179, 123	perovskin B
**11**	16.00	287.1332	$C_{18}H_{22}O_3$	-0.96	269, 217	perovskin C
**12**	16.39	241.1584	$C_{17}H_{20}O$			12-hydroxy-16,17-bis-nor-simonellite
**13**	16.67	317.2102	$C_{20}H_{29}O_3$	-2.78	299, 271, 199	demethylsalvican-11,12-dione
**14**	16.69	273.1844	$C_{18}H_{24}O_2$	-1.89		przewalskin
**15**	17.52	313.1430	$C_{19}H_{20}O_4$	-1.26	295, 267, 243	1β-hydroxycryptotanshinone
**16**	18.07	271.1684	$C_{18}H_{22}O_2$	-3.05	229, 201	perovskin D
**17**	19.32	303.1945	$C_{20}H_{30}O_2$		203, 189, 161	pisiferol
**18**	20.02	297.1481	$C_{19}H_{20}O_3$	-1.42	279, 251	cryptotanshinone
**19**	20.62	301.2154	$C_{20}H_{28}O_2$	-2.71	259, 163, 173	11-hydroxyabieta-8,11,13-trien-7-one
**20**	22.11	329.1739	$C_{20}H_{24}O_4$	-2.66	315, 297, 241	12-*O*-methyl-miltiorin D
**21**	23.26	287.1633	$C_{18}H_{22}O_3$	-3.033	269	epicryptoacetalide
**22**	23.34	315.1945	$C_{20}H_{27}O_3$	-2.92	205, 179	montbretrol
**23**	24.97	317.2101	$C_{20}H_{28}O_3$	-3.19	299, 27	6-deoxy-salviphlomone
**24**	25.25	299.1630	$C_{19}H_{23}O_3$	-3.71		miltiodiol
**25**	26.19	269.1528	$C_{18}H_{21}O_2$	-0.78		salviolone

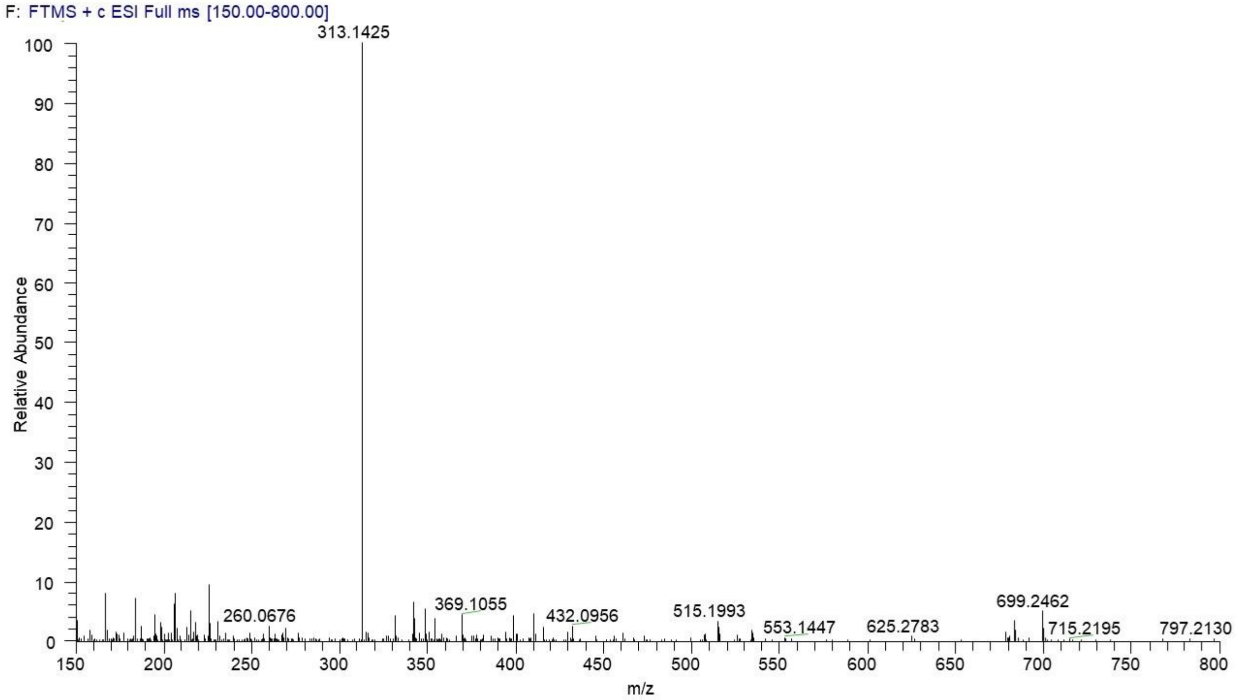

Figure S1. ESI/LTQOrbitrap spectrum of compound **2**, in positive ion mode.

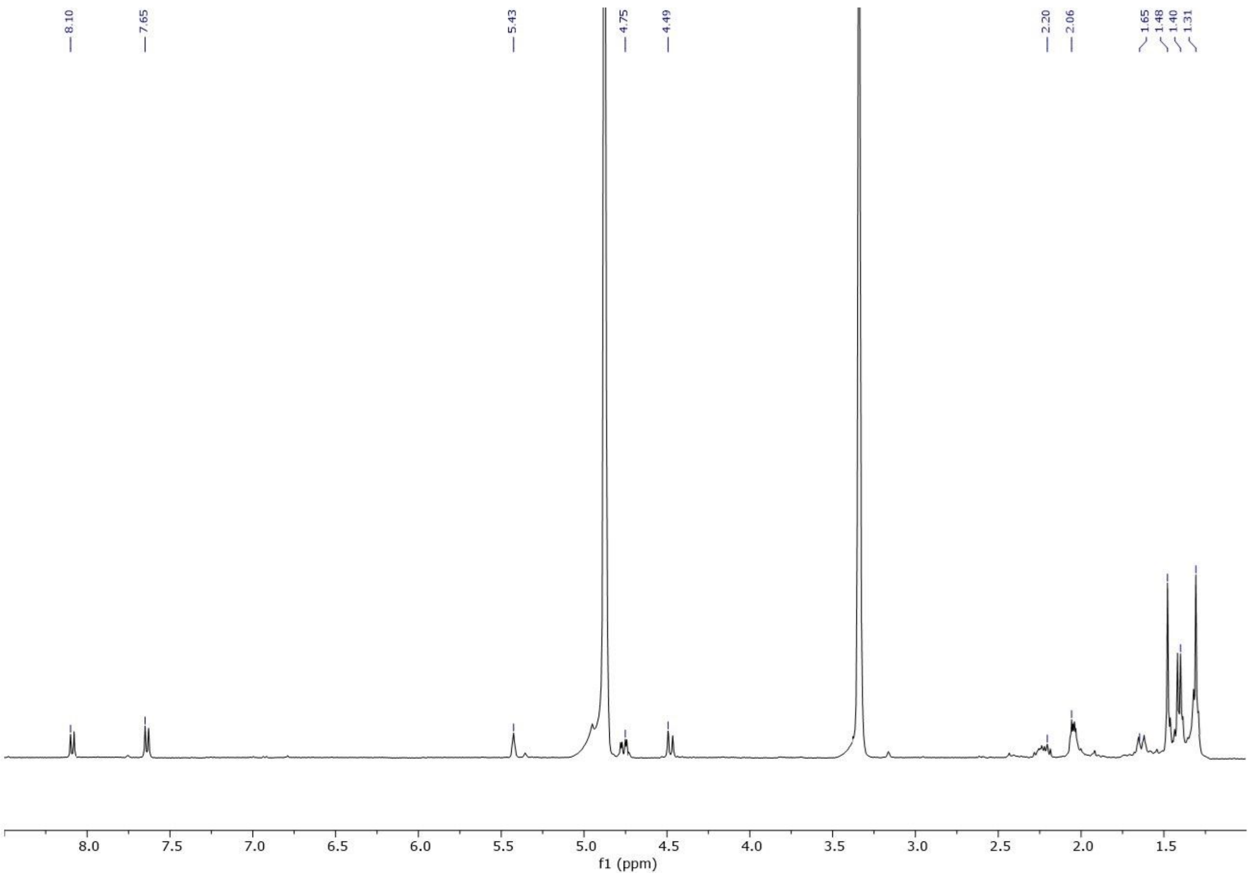

Figure S2. ^{1}H NMR Spectrum (600 MHz, CD_3OD) of compound **2**.

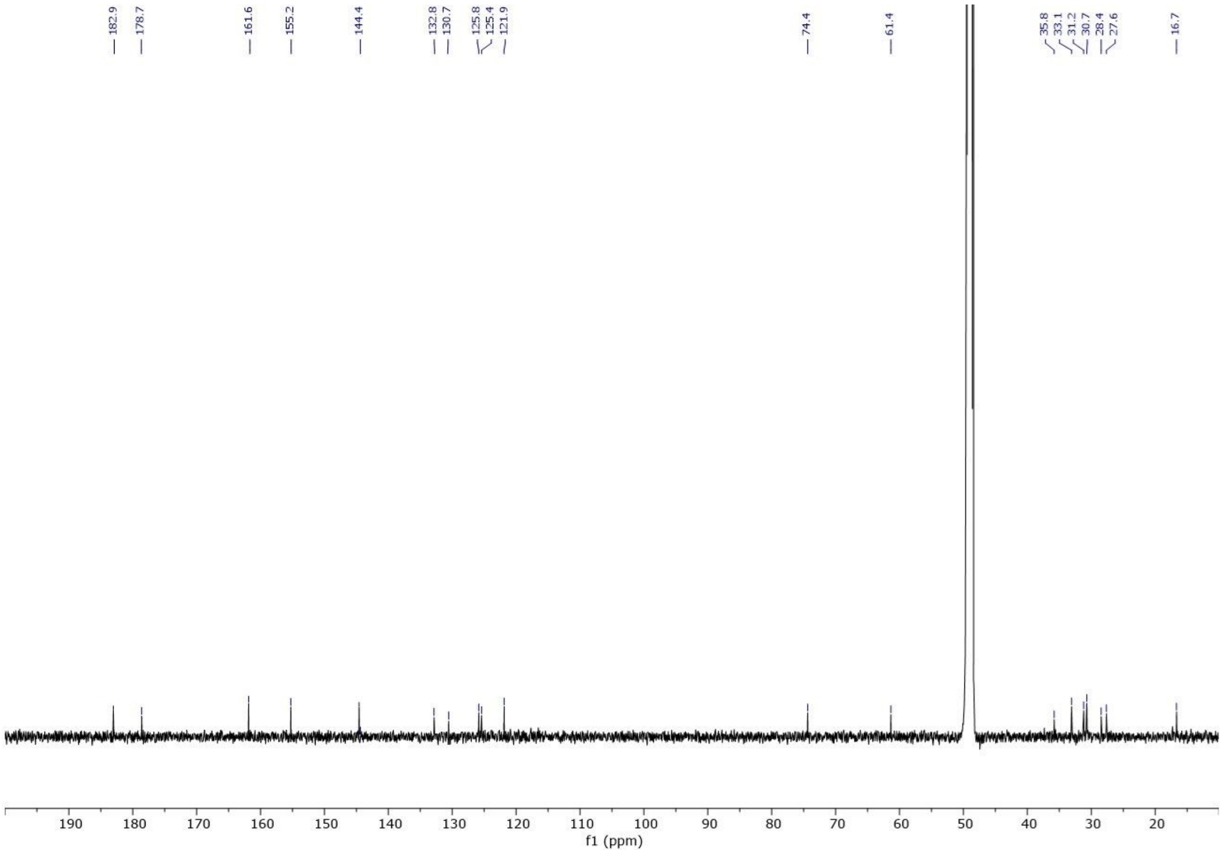

Figure S3. ^{13}C Spectrum (150 MHz, CD_3OD) of compound **2**.

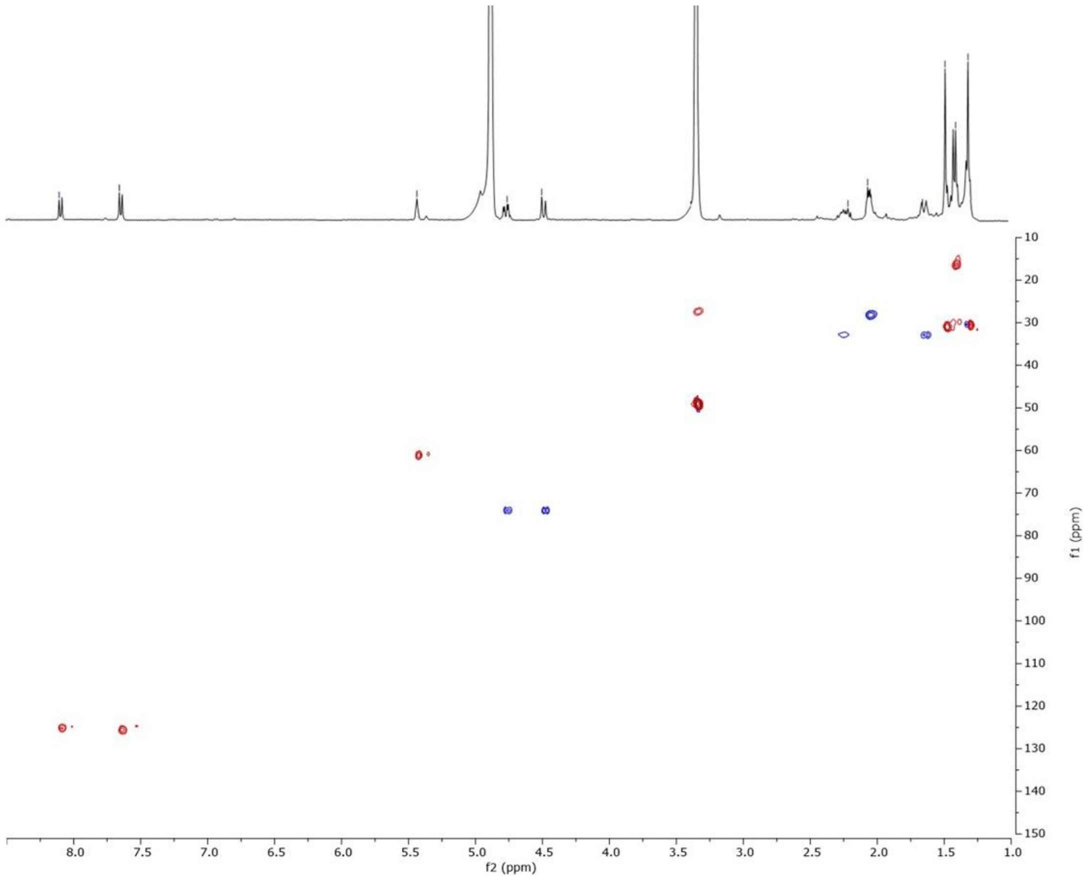

Figure S4. HSQC Spectrum (CD_3OD) of compound **2**.

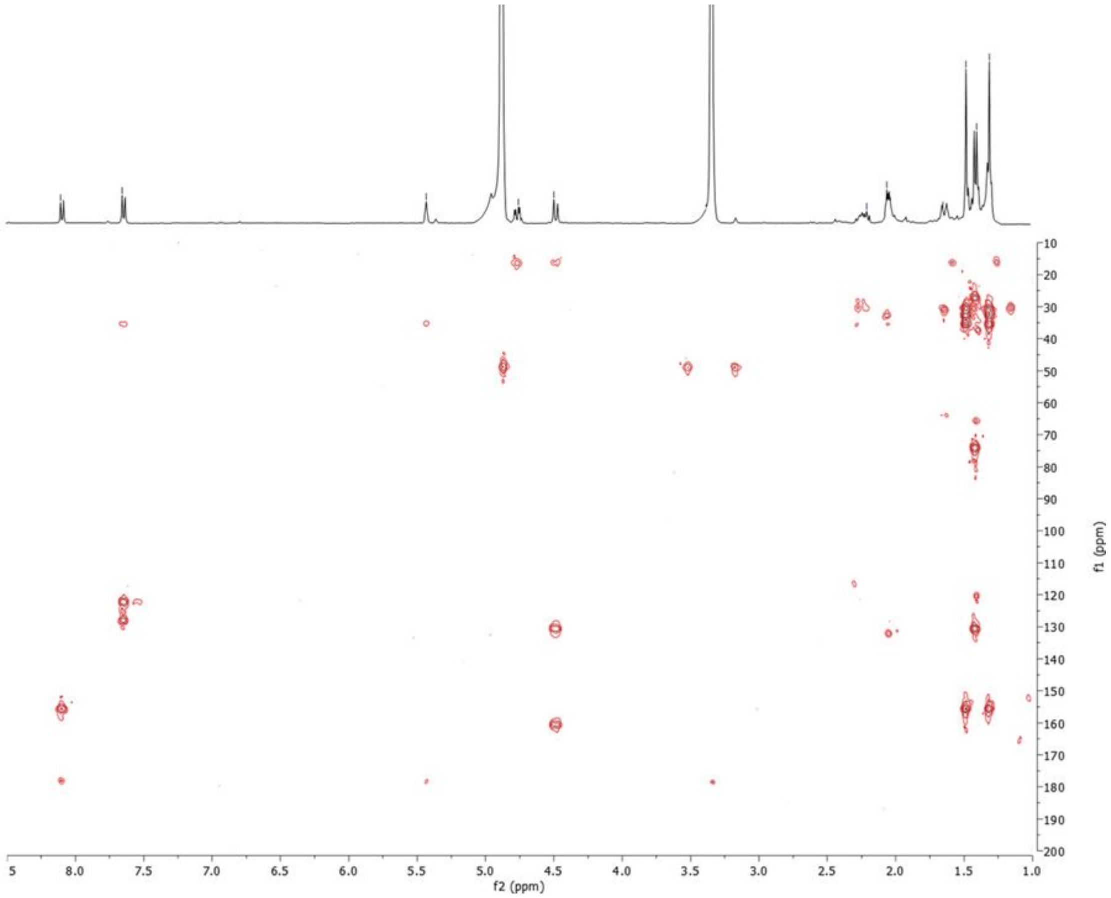

Figure S5. HMBC Spectrum (CD_3OD) of compound **2**.

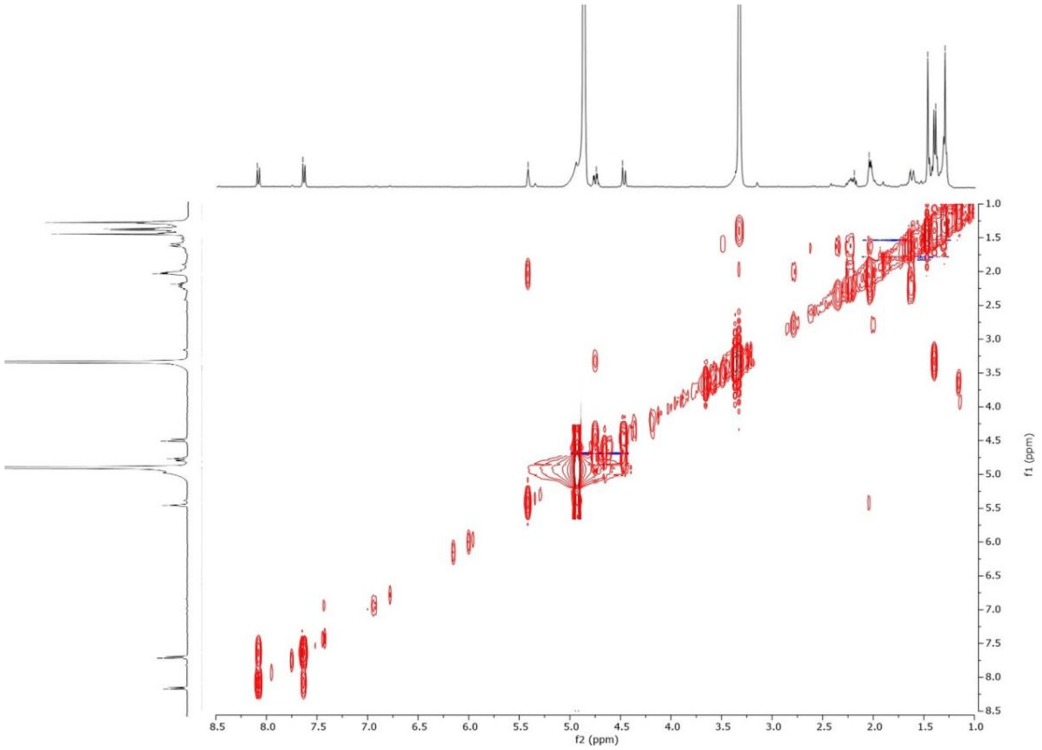

Figure S6. COSY Spectrum (CD_3OD) of compound **2**.

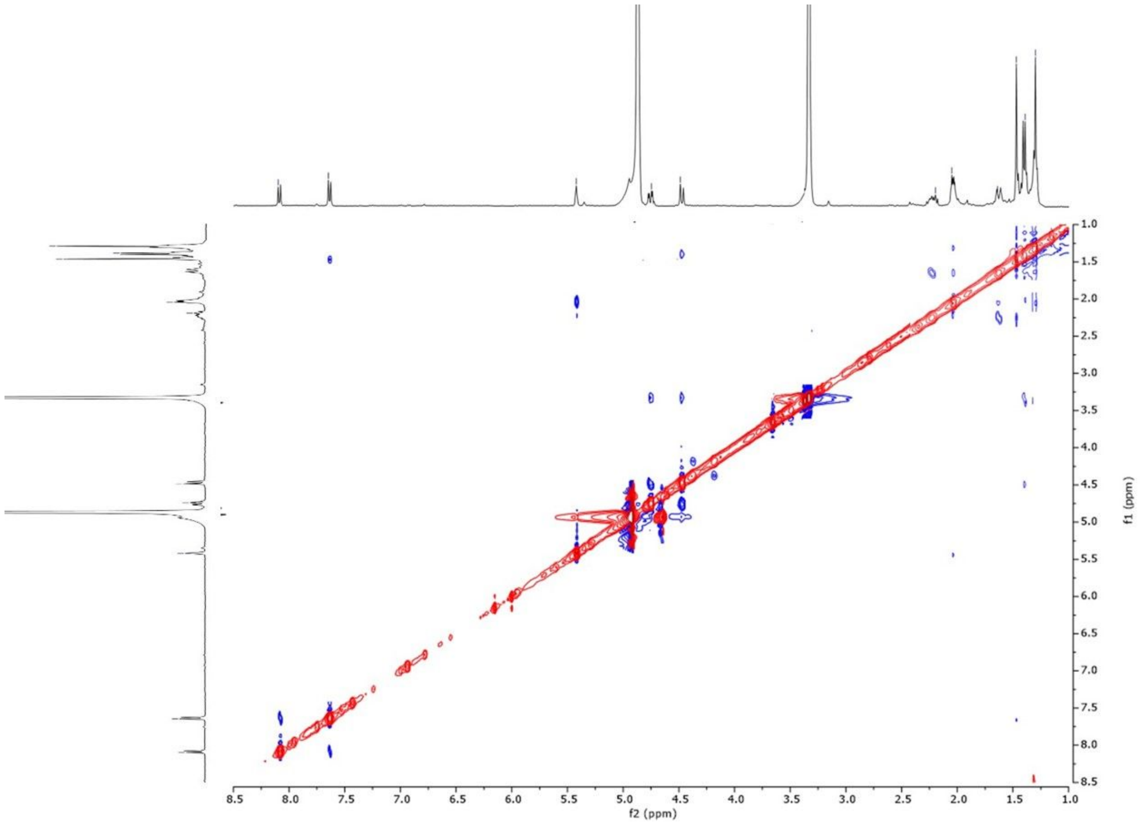

Figure S7. ROESY Spectrum (CD_3OD) of compound **2**.

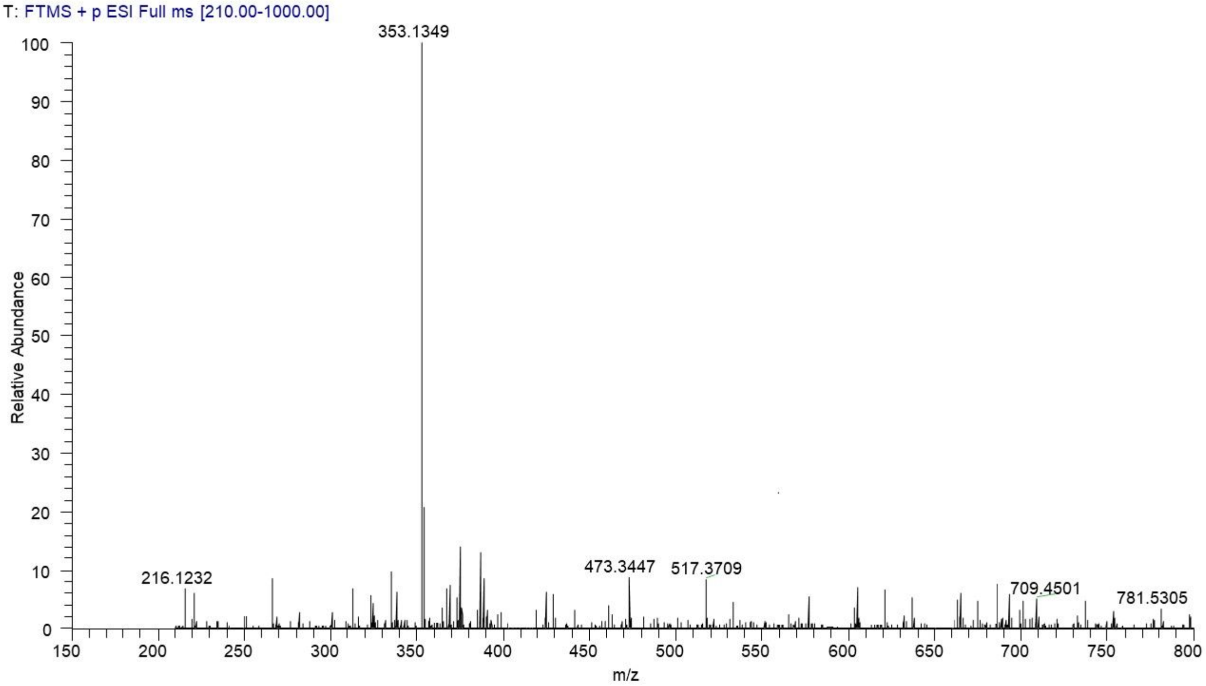

Figure S8. ESI/LTQOrbitrap spectrum of compound **9**, in positive ion mode.

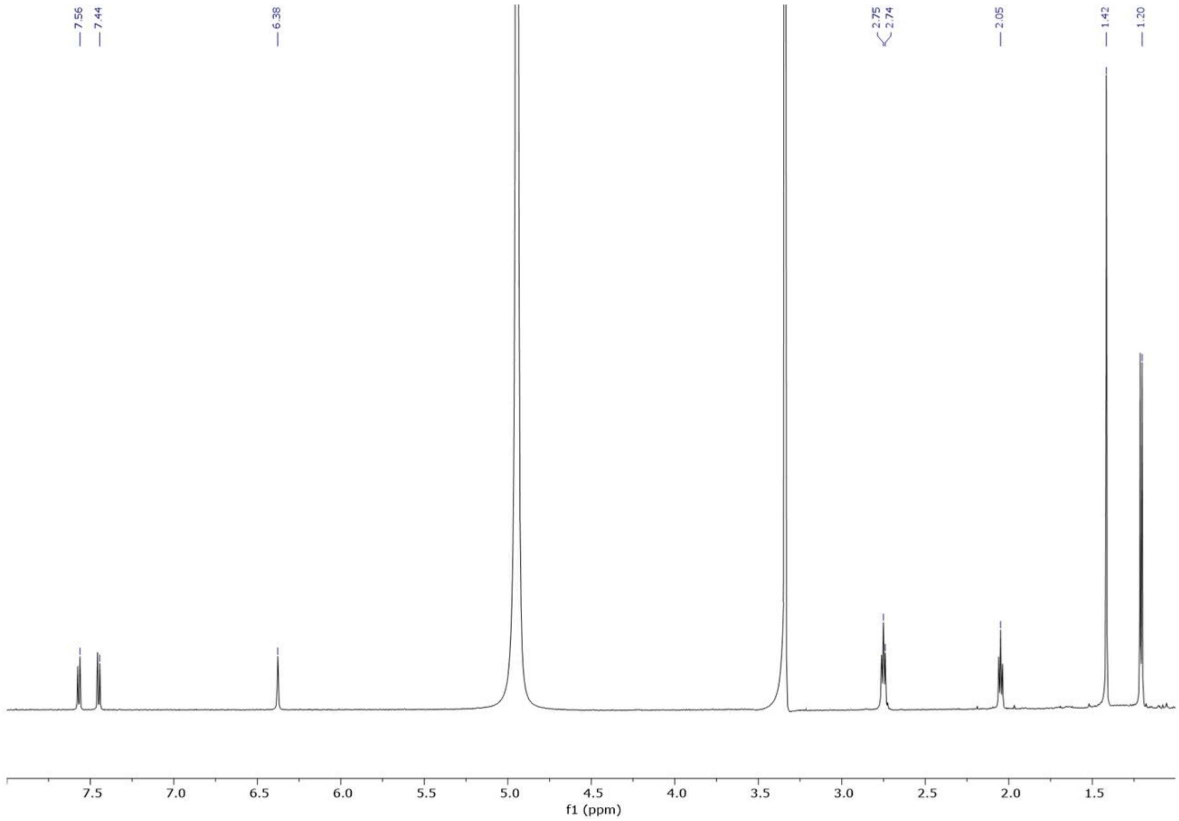

Figure S9. ^{1}H NMR Spectrum (600 MHz, CD_3OD) of compound **9**.

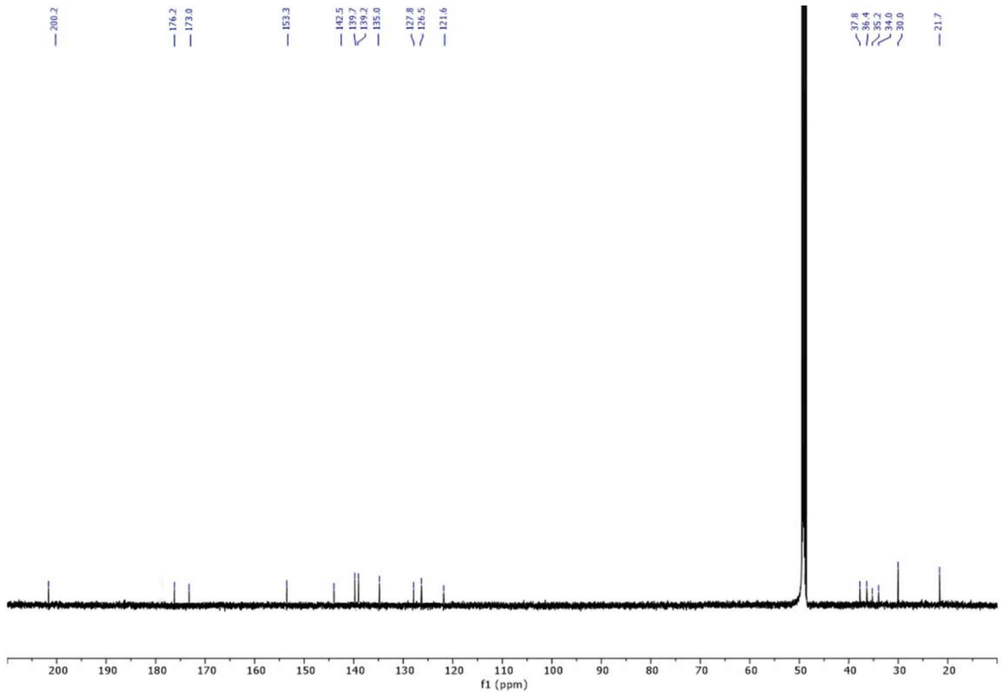

Figure S10. ^{13}C Spectrum (150 MHz, CD_3OD) of compound **9**.

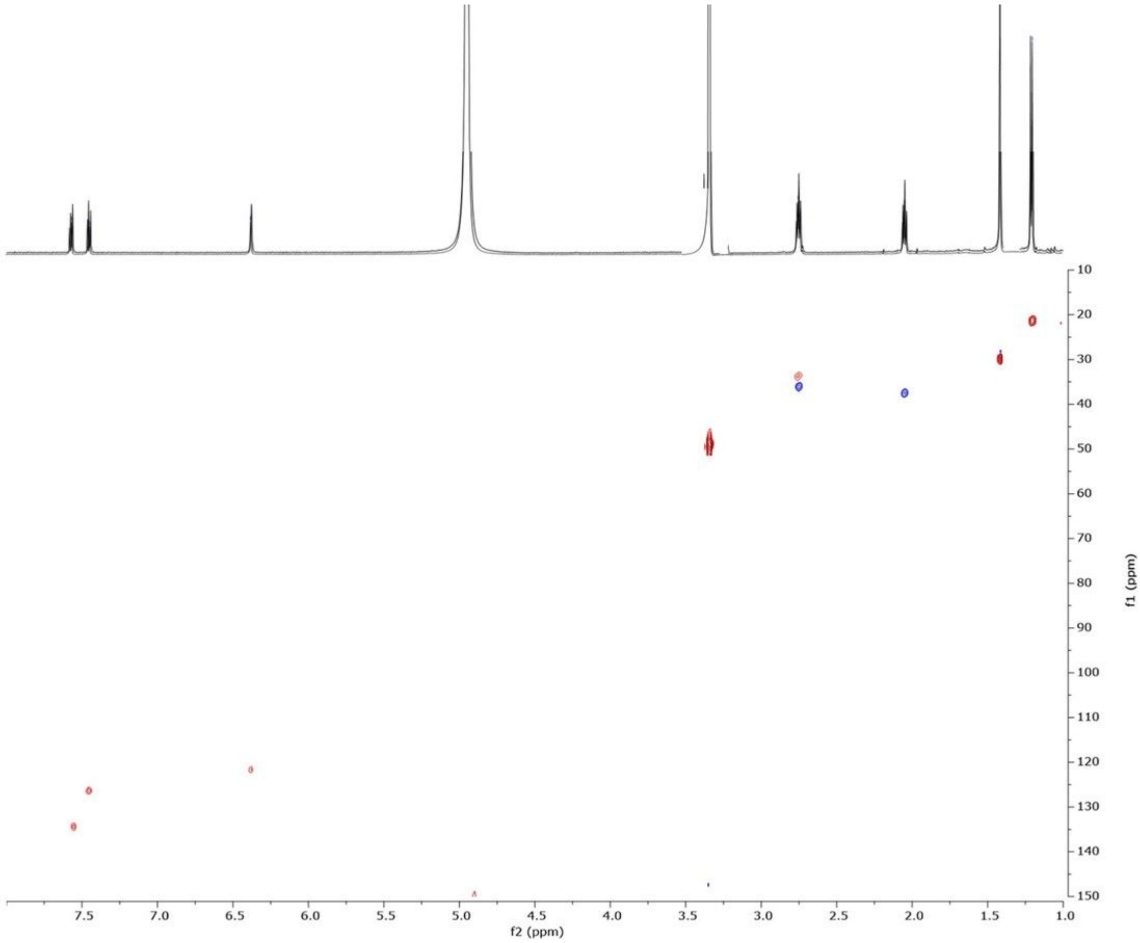

Figure S11. HSQC Spectrum (CD_3OD) of compound **9**.

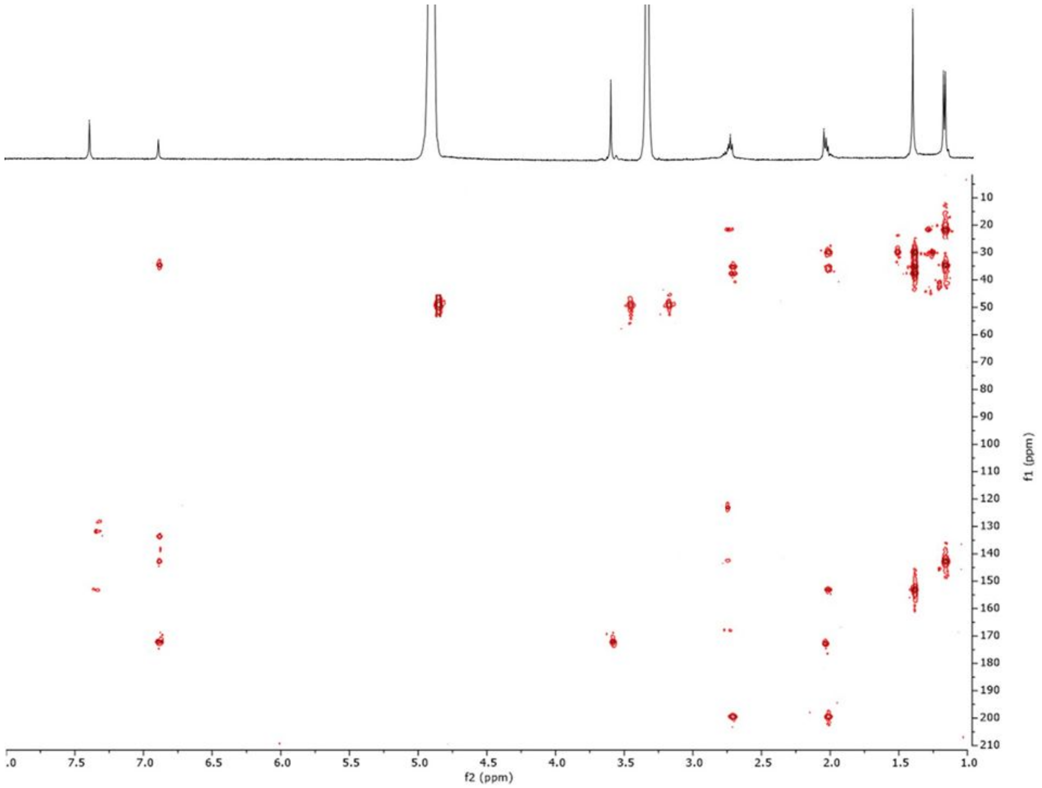

Figure S12. HMBC Spectrum (CD_3OD) of compound **9**.

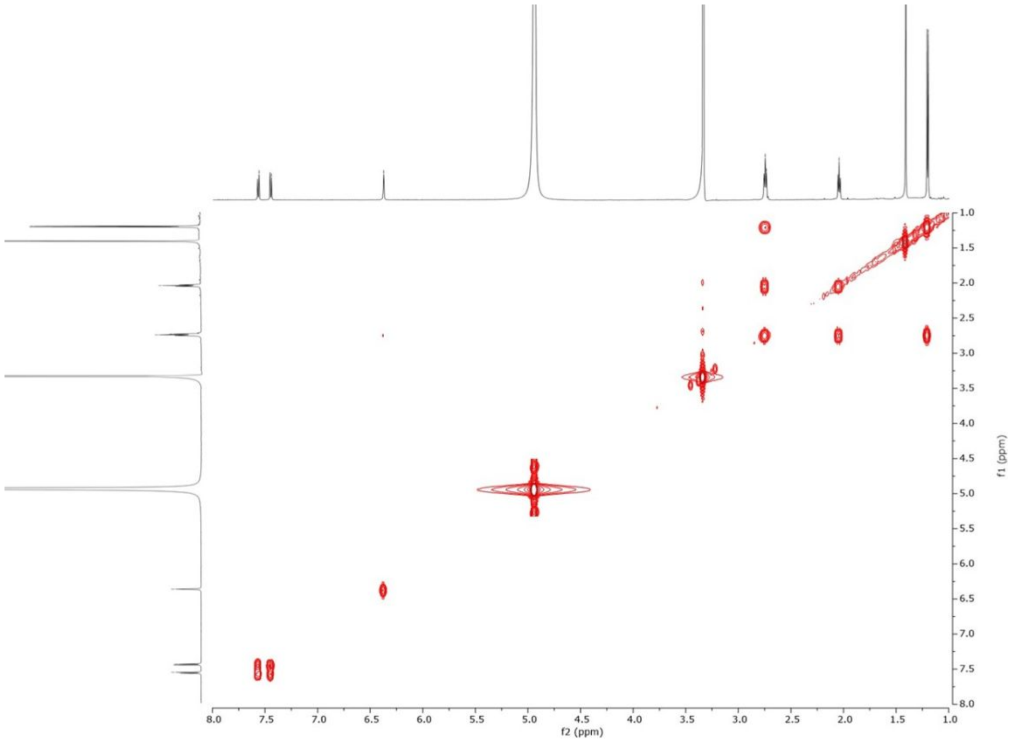

Figure S13. COSY Spectrum (CD_3OD) of compound **9**.

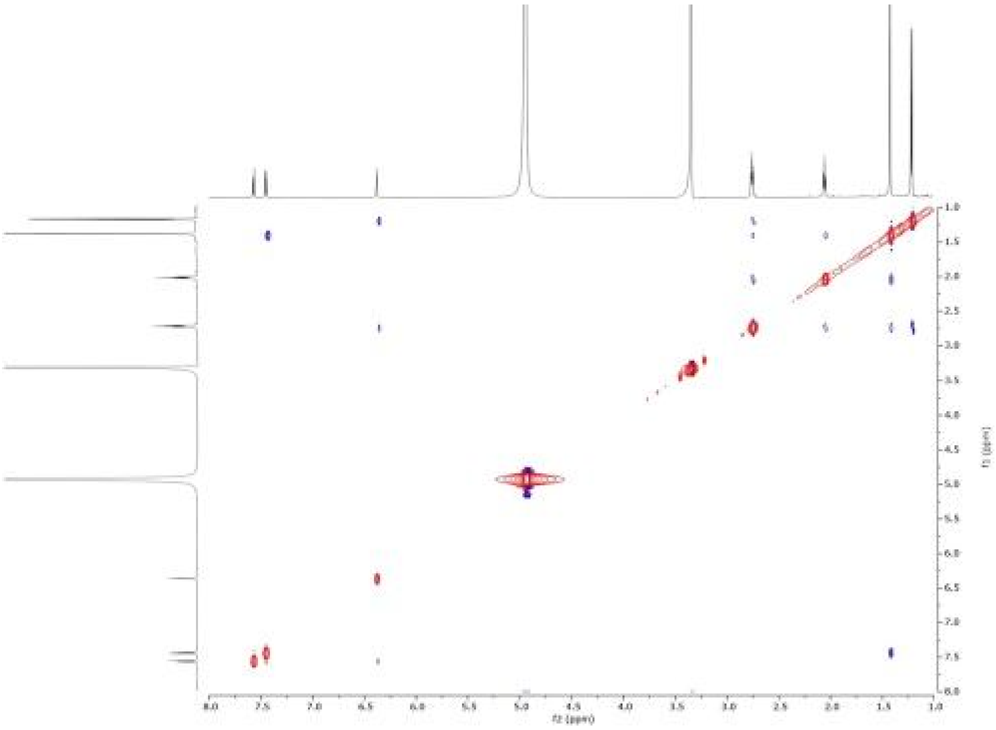

Figure S14. ROESY Spectrum (CD_3OD) of compound **9**

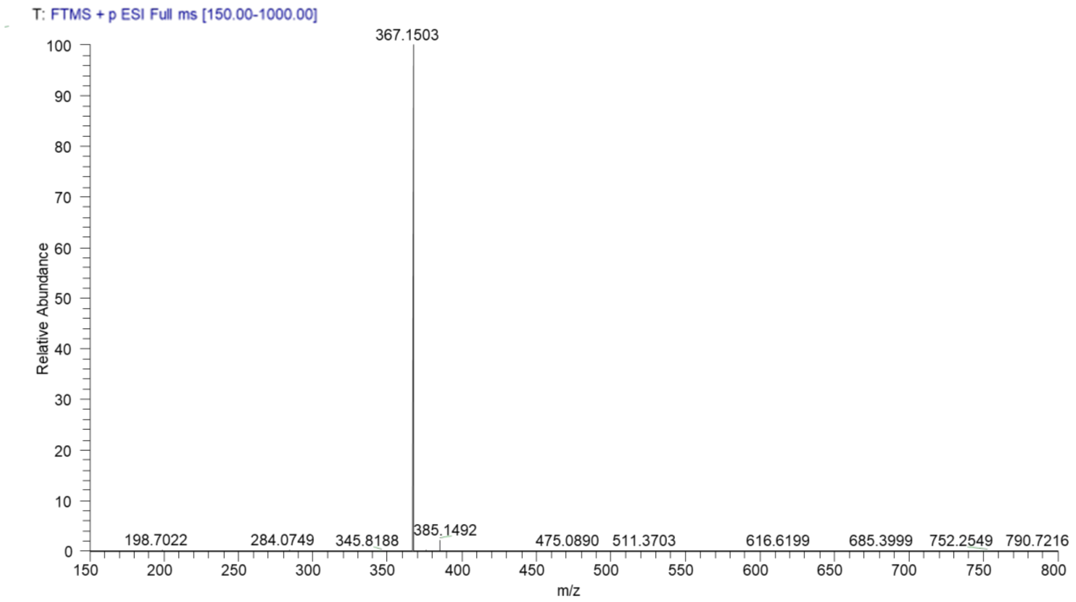

Figure S15. ESI/LTQOrbitrap spectrum of compound **10**, in positive ion mode.

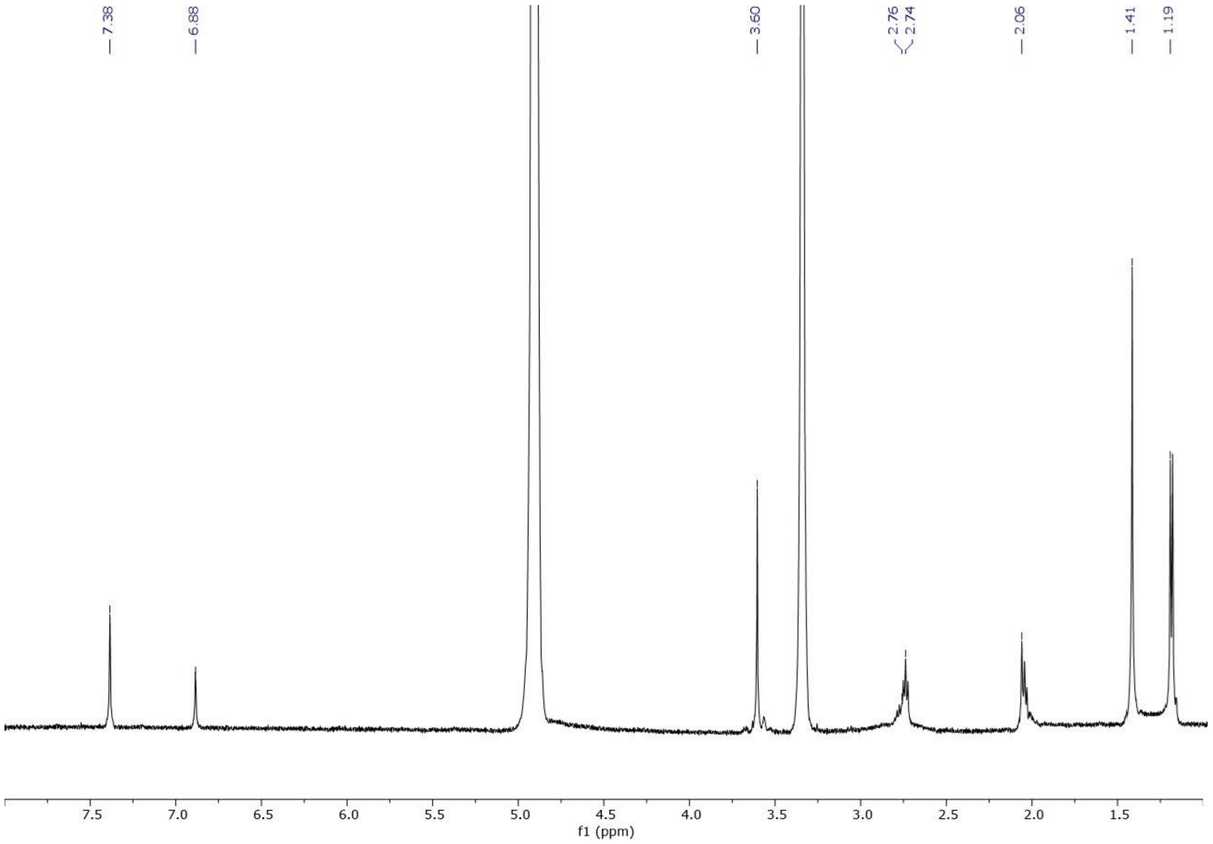

Figure S16.^{1}H NMR Spectrum (600 MHz, CD_3OD) of compound **10**.

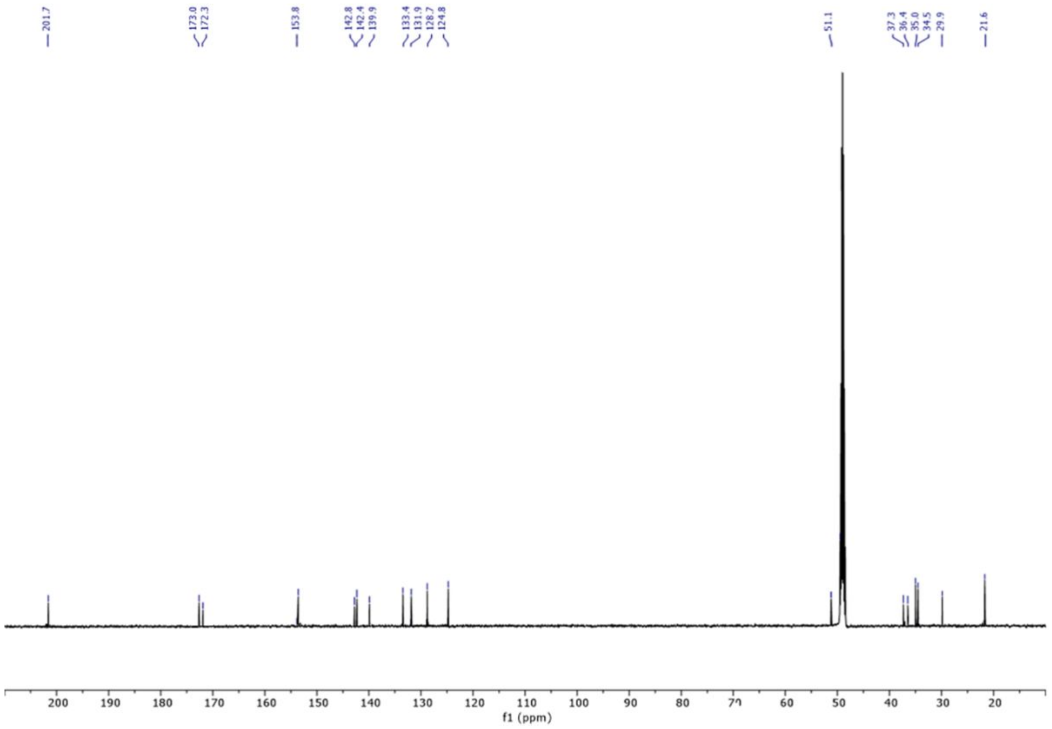

Figure S17. ^{13}C Spectrum (150 MHz, CD_3OD) of compound **10**.

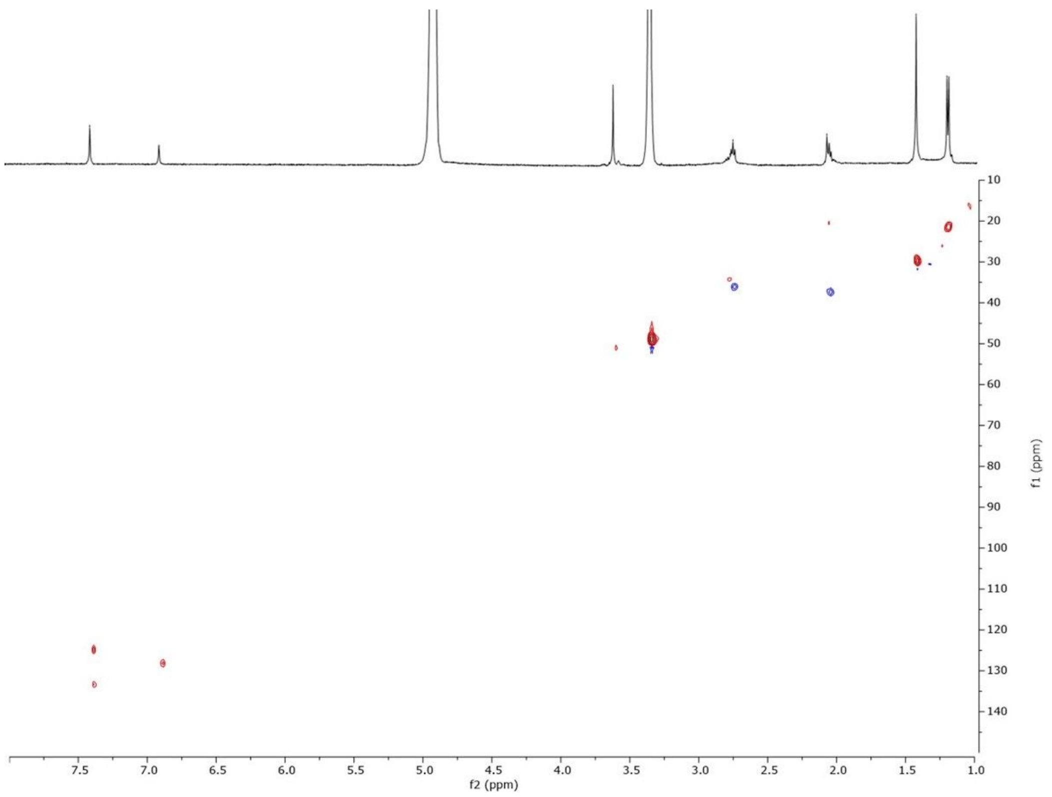

Figure S18. HSQC Spectrum (CD_3OD) of compound **10**.

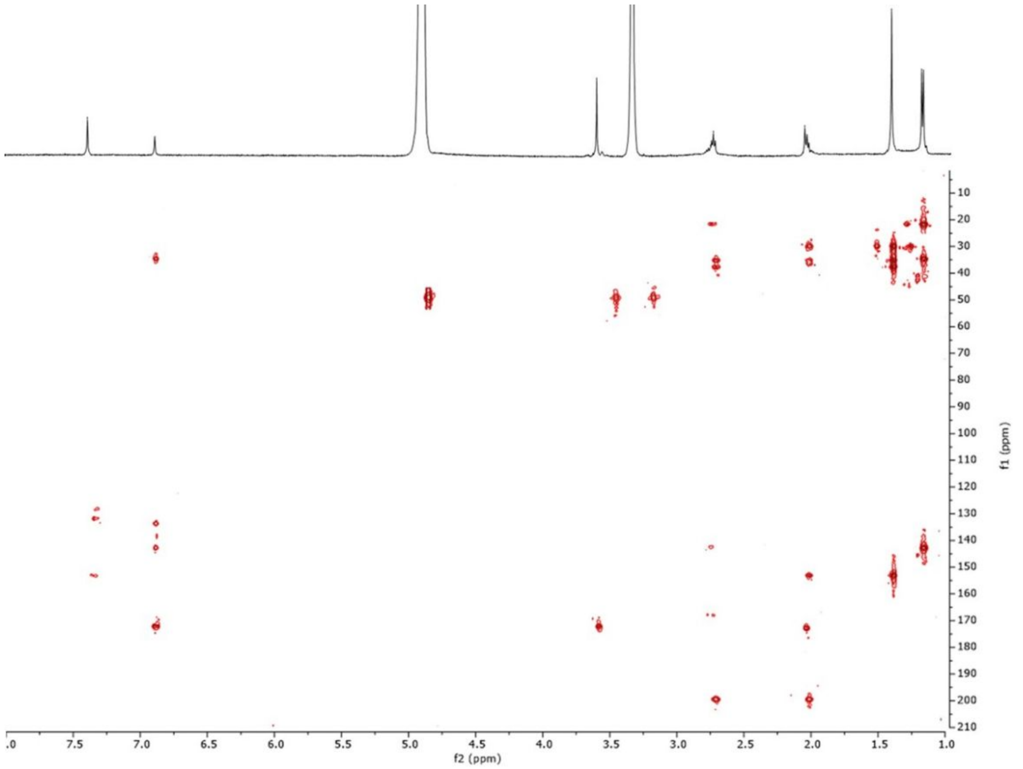

Figure S19. HMBC Spectrum (CD_3OD) of compound **10**.

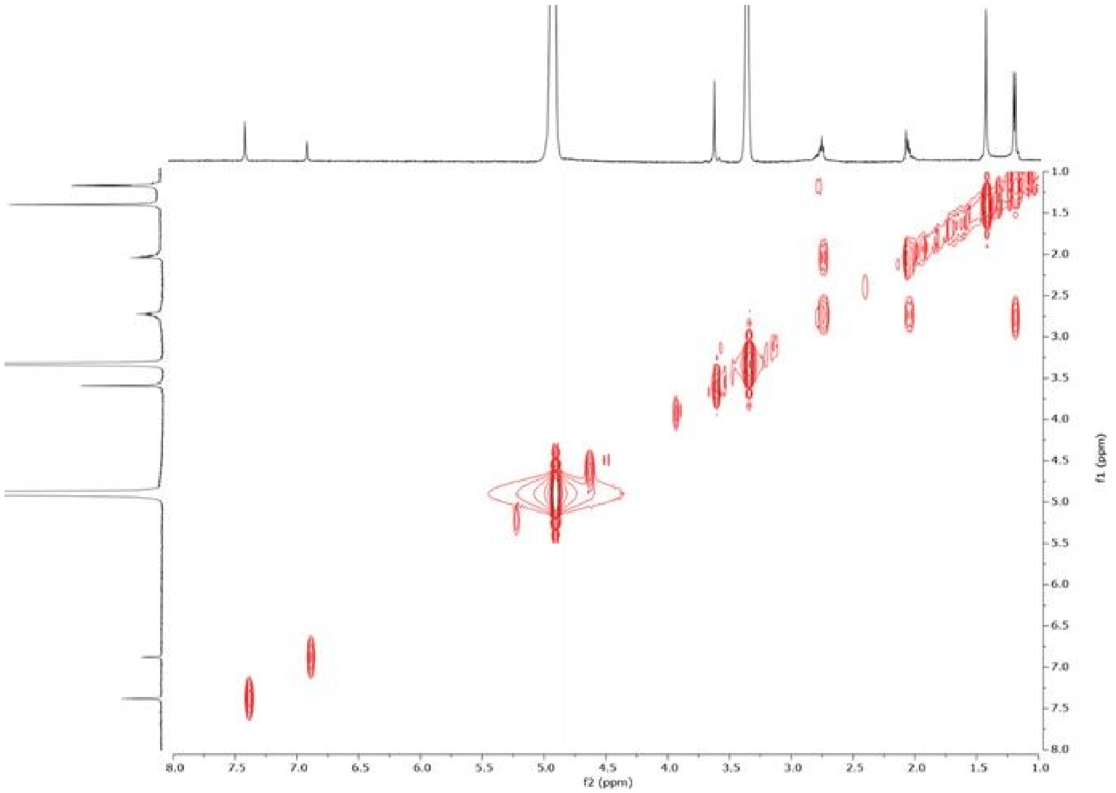

Figure S20. COSY Spectrum (CD_3OD) of compound **10**.

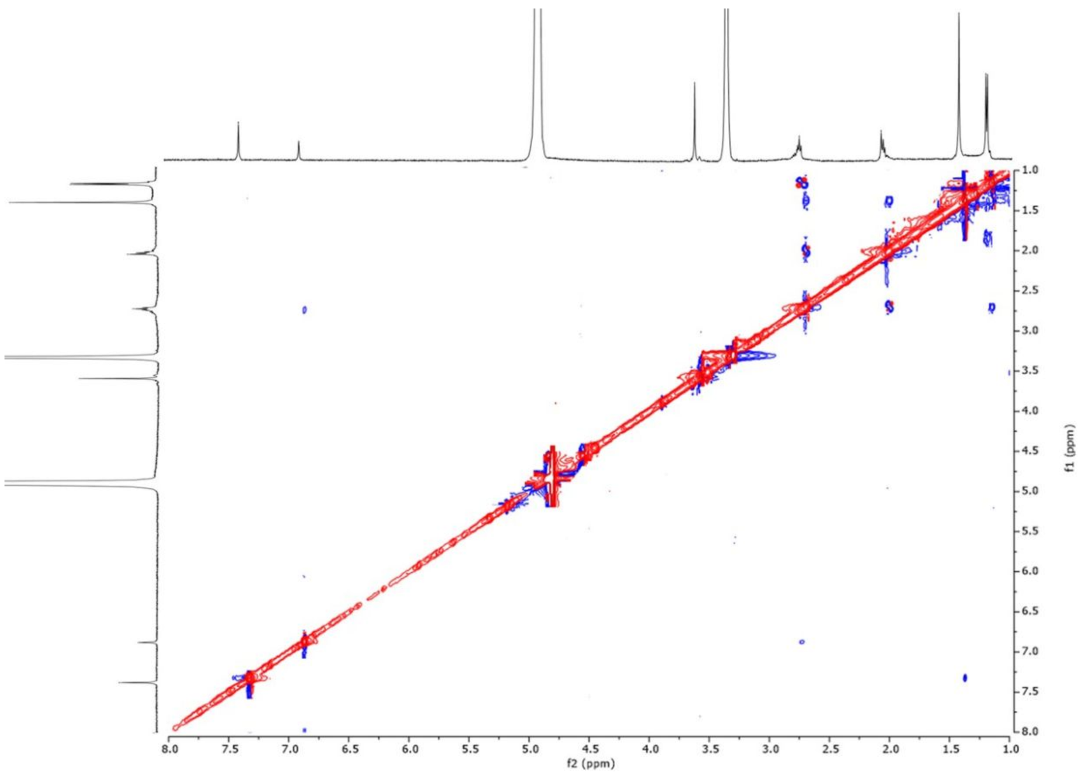

Figure S21. ROESY Spectrum (CD_3OD) of compound **10**.

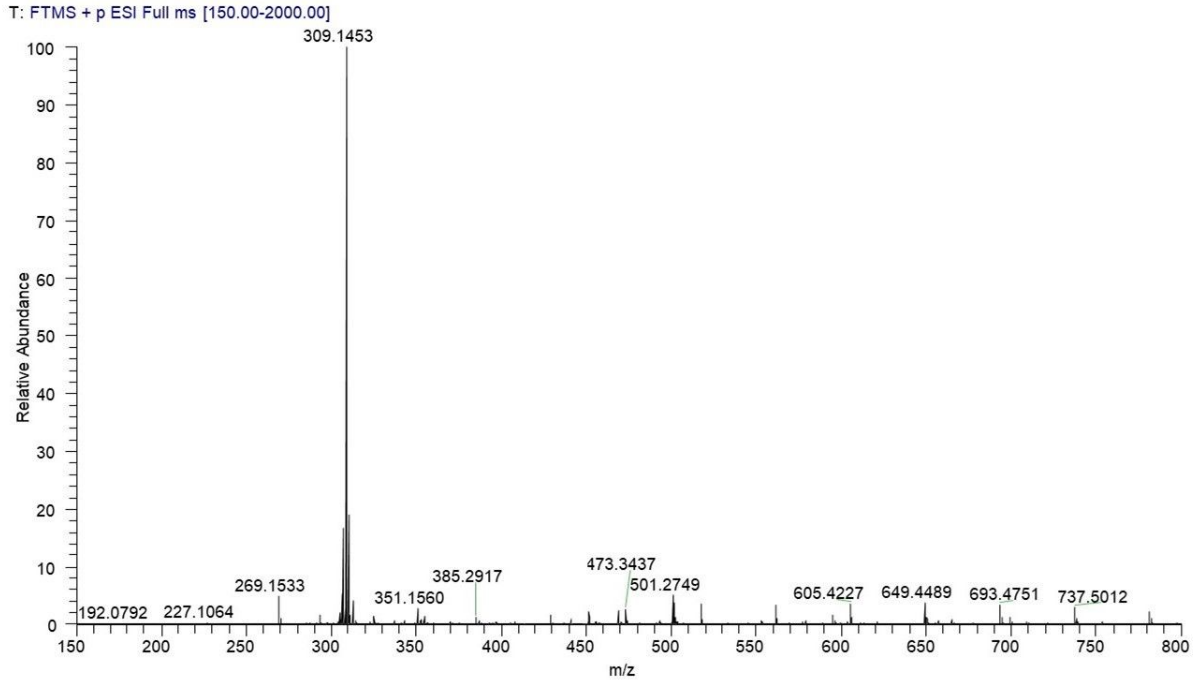

Figure S22. ESI/LTQOrbitrap spectrum of compound **11**, in negative ion mode.

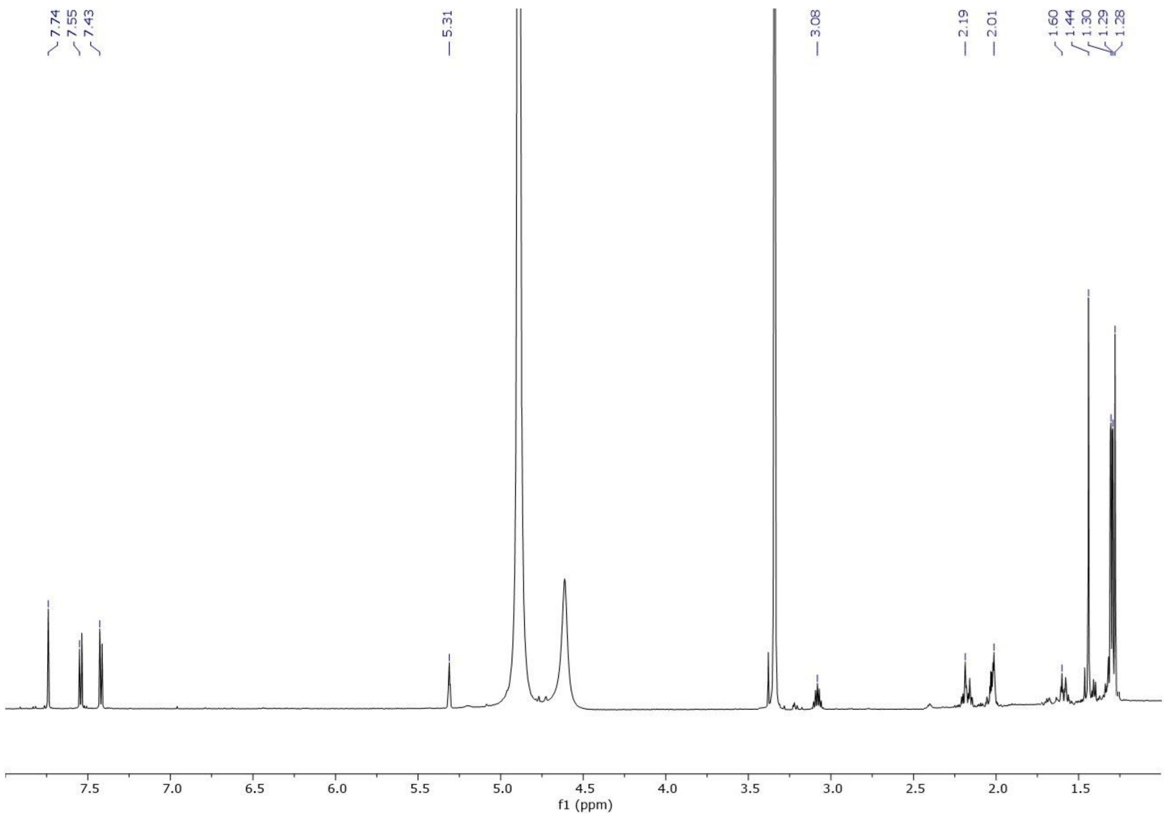

Figure S23. ^{1}H NMR Spectrum (600 MHz, CD_3OD) of compound **11**.

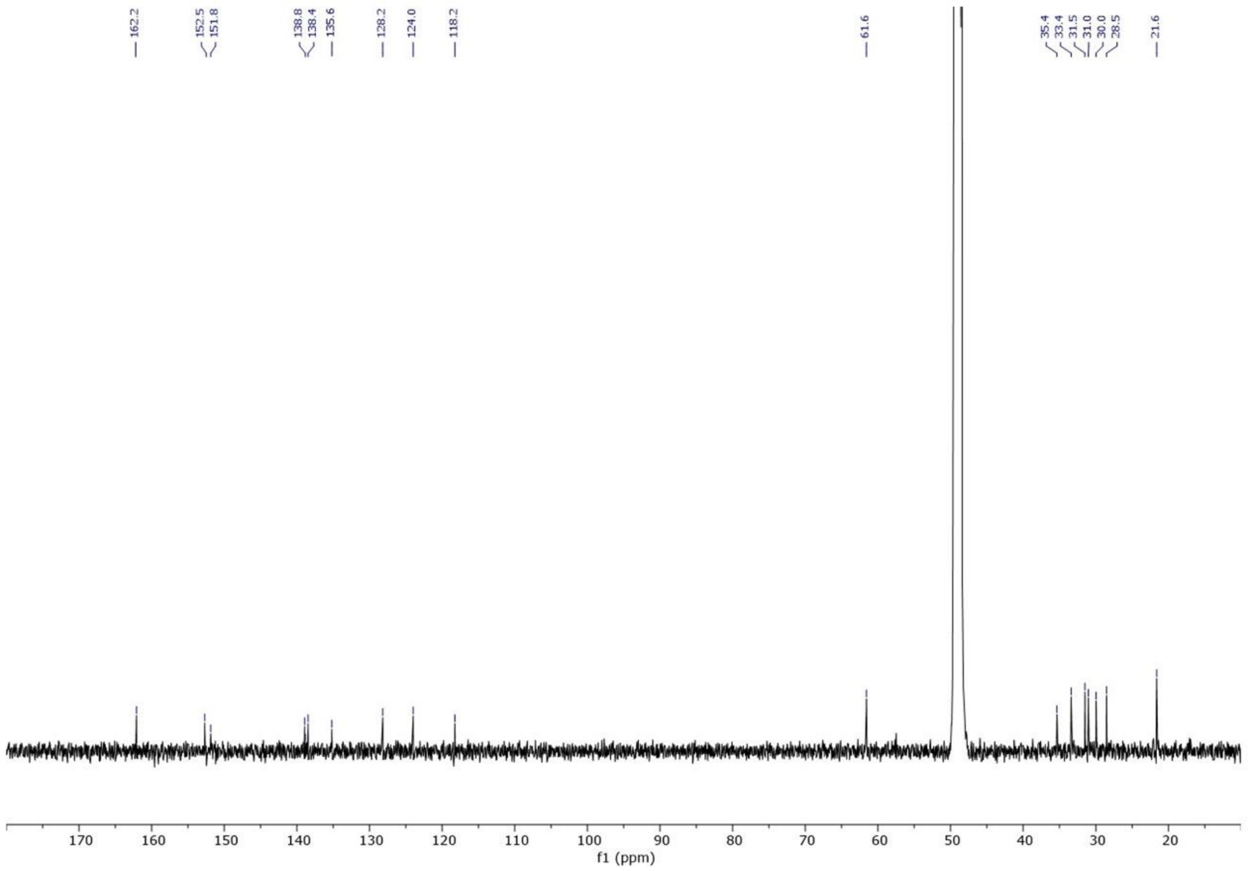

Figure S24. ^{13}C Spectrum (150 MHz, CD_3OD) of compound **11**.

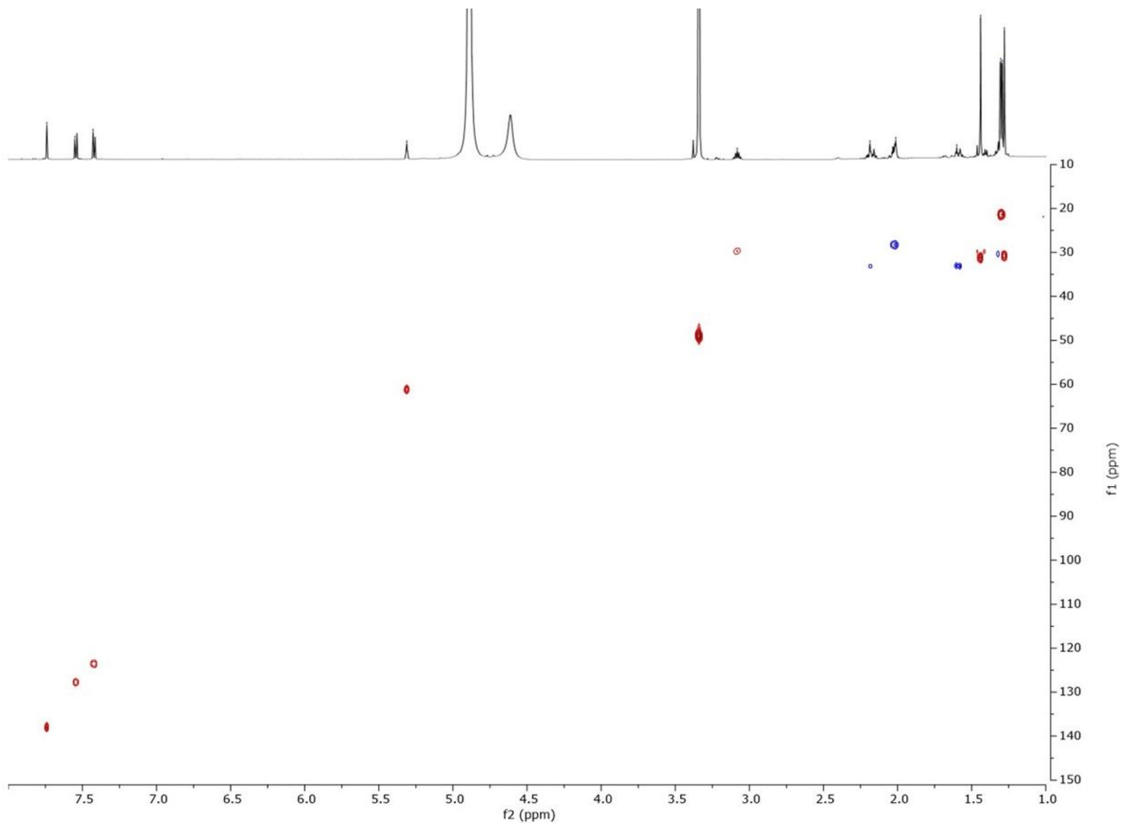

Figure S25. HSQC Spectrum (CD_3OD) of compound **11**.

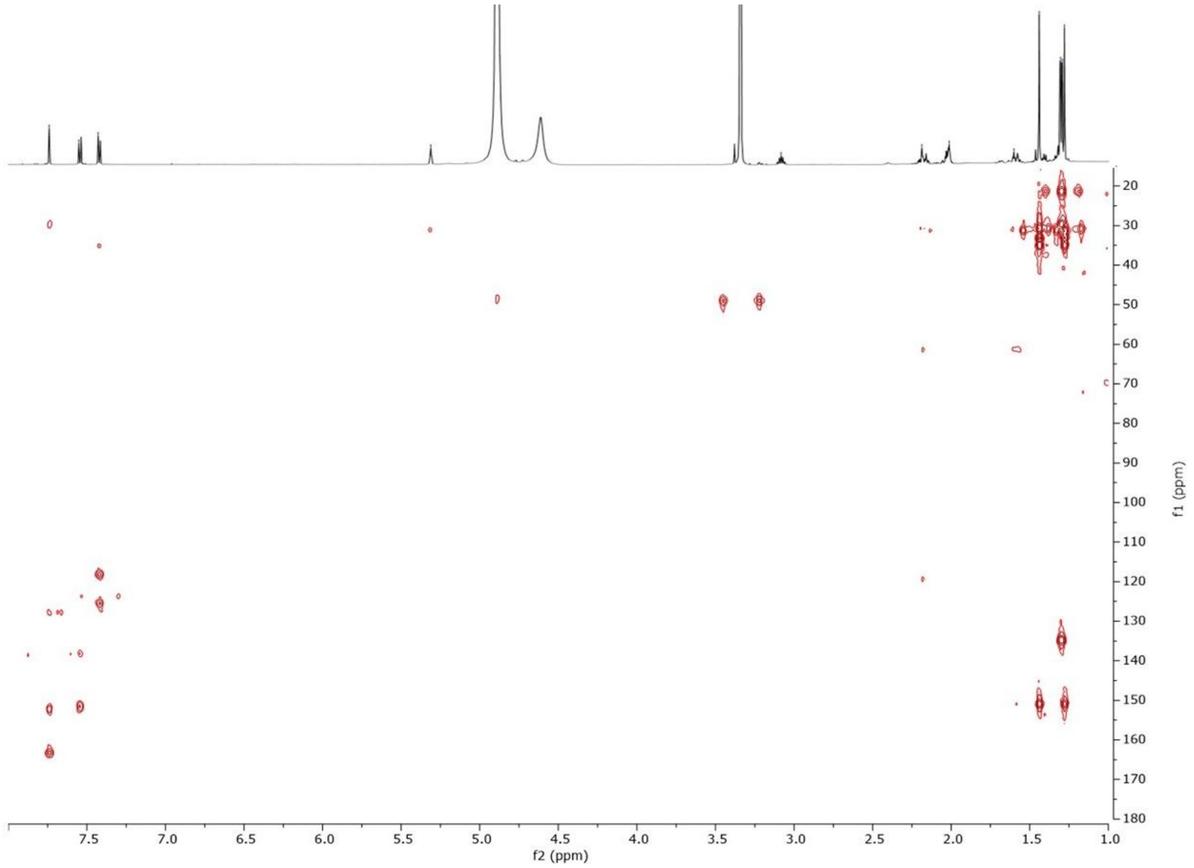

Figure S26. HMBC Spectrum (CD_3OD) of compound **11**.

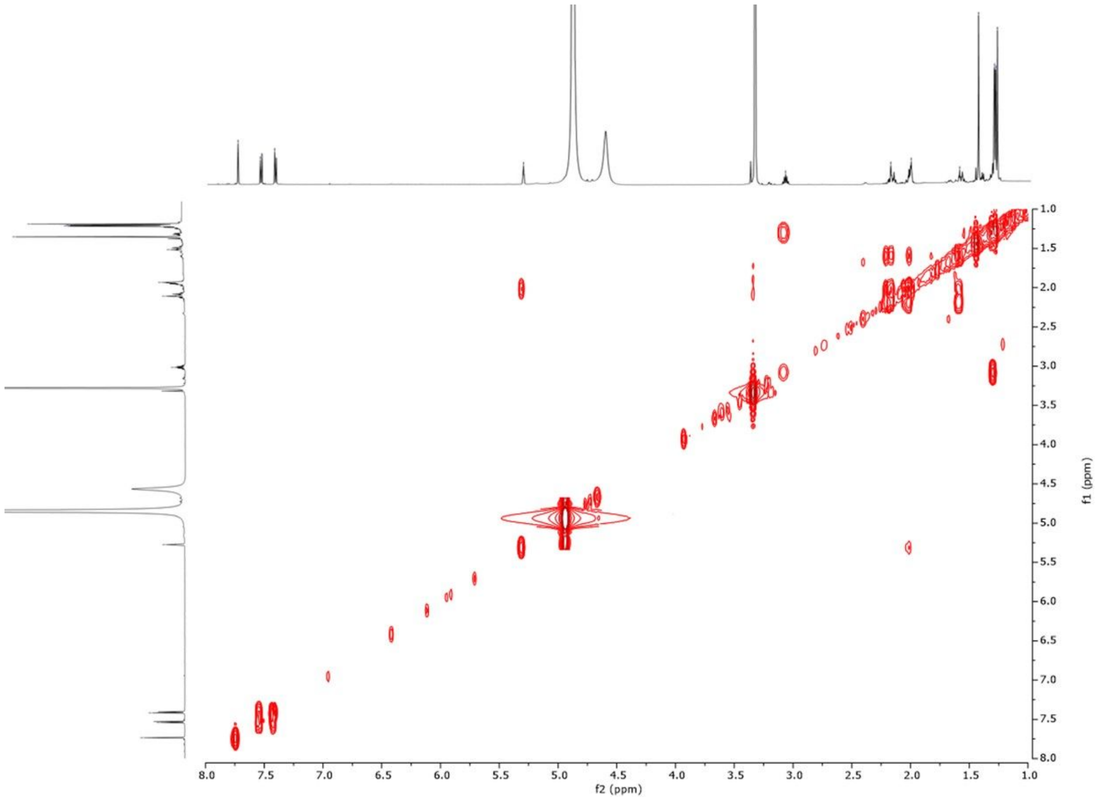

Figure S27. COSY Spectrum (CD_3OD) of compound **11.**

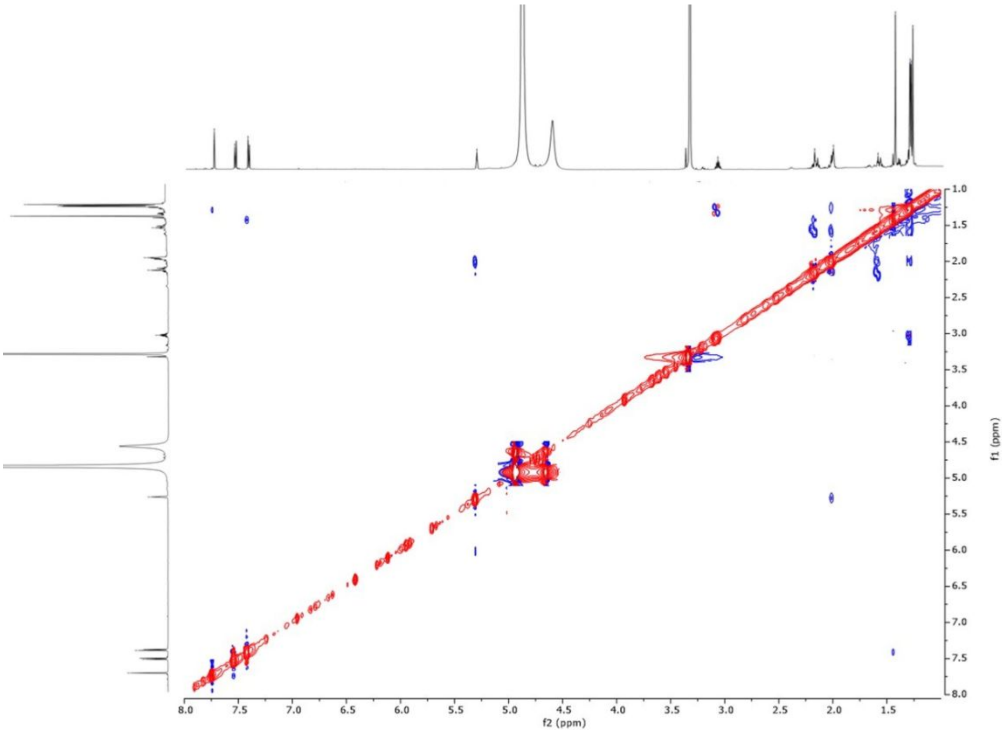

Figure S28. ROESY Spectrum (CD_3OD) of compound **11.**

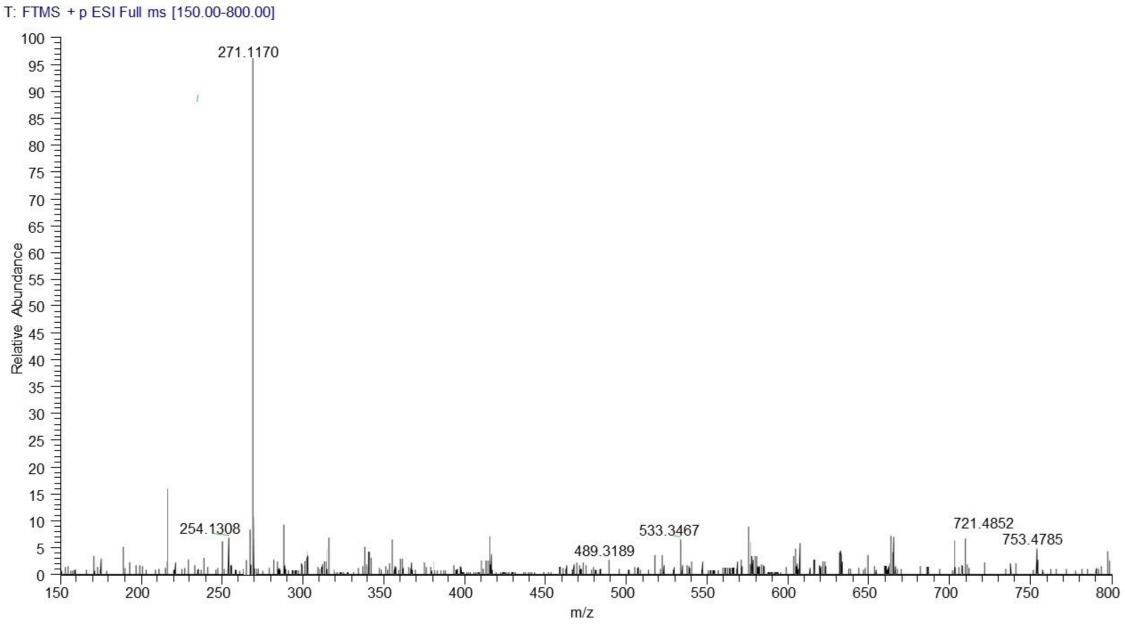

Figure S29. ESI/LTQOrbitrap spectrum of compound **16**, in negative ion mode.

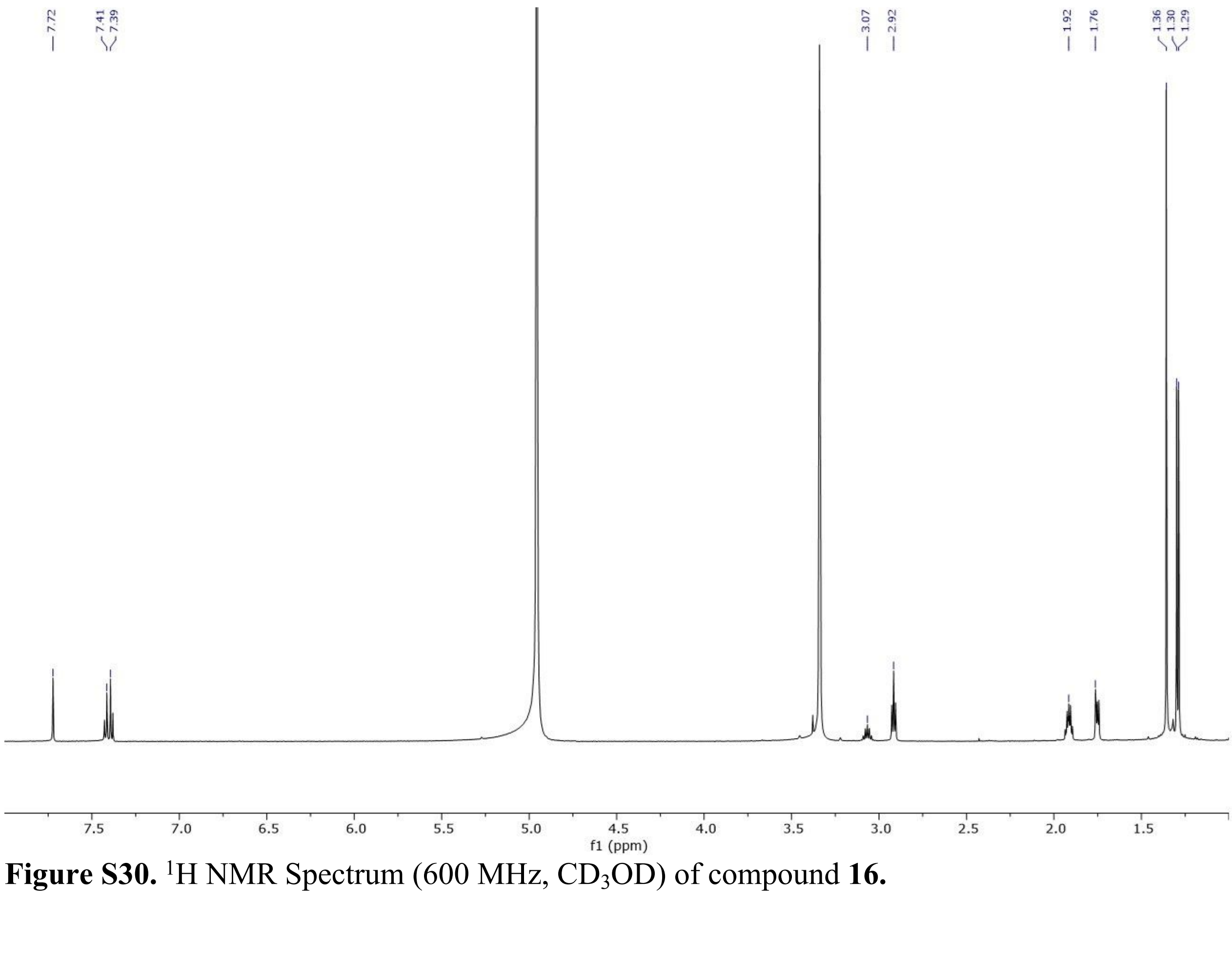

Figure S30. ^{1}H NMR Spectrum (600 MHz, CD_3OD) of compound **16.**

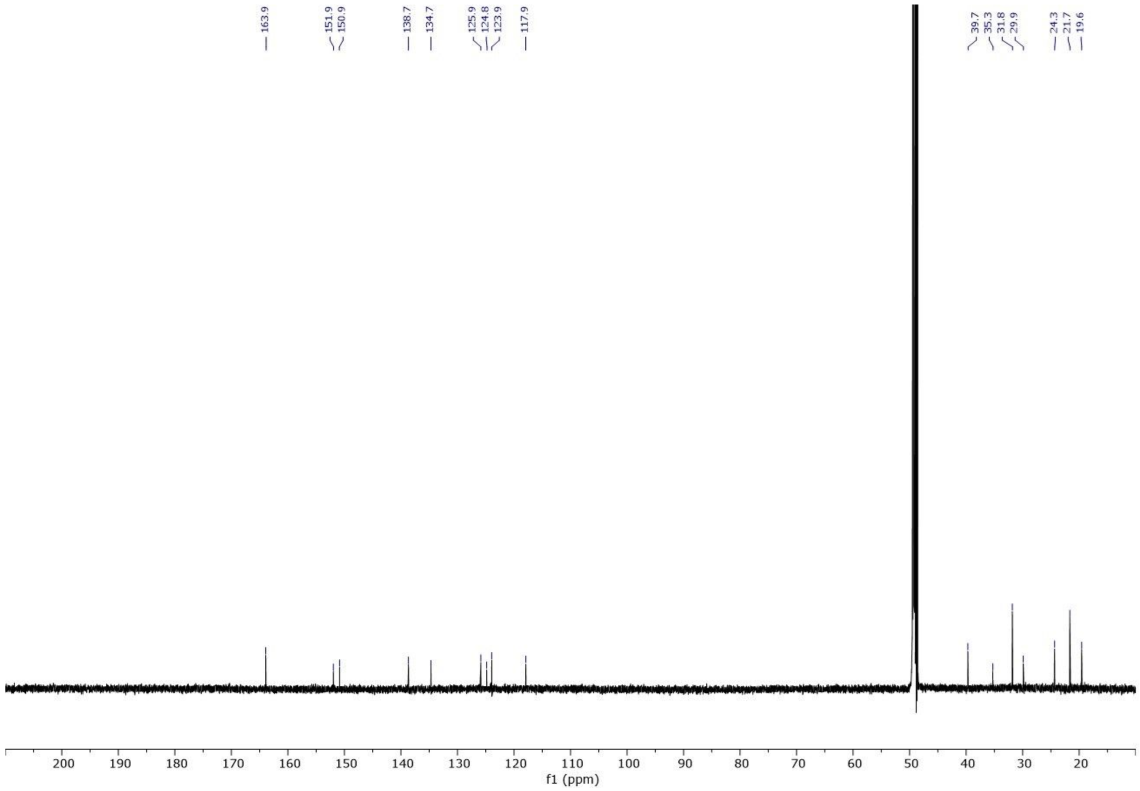

Figure S31. ^{13}C Spectrum (150 MHz, CD_3OD) of compound **16.**

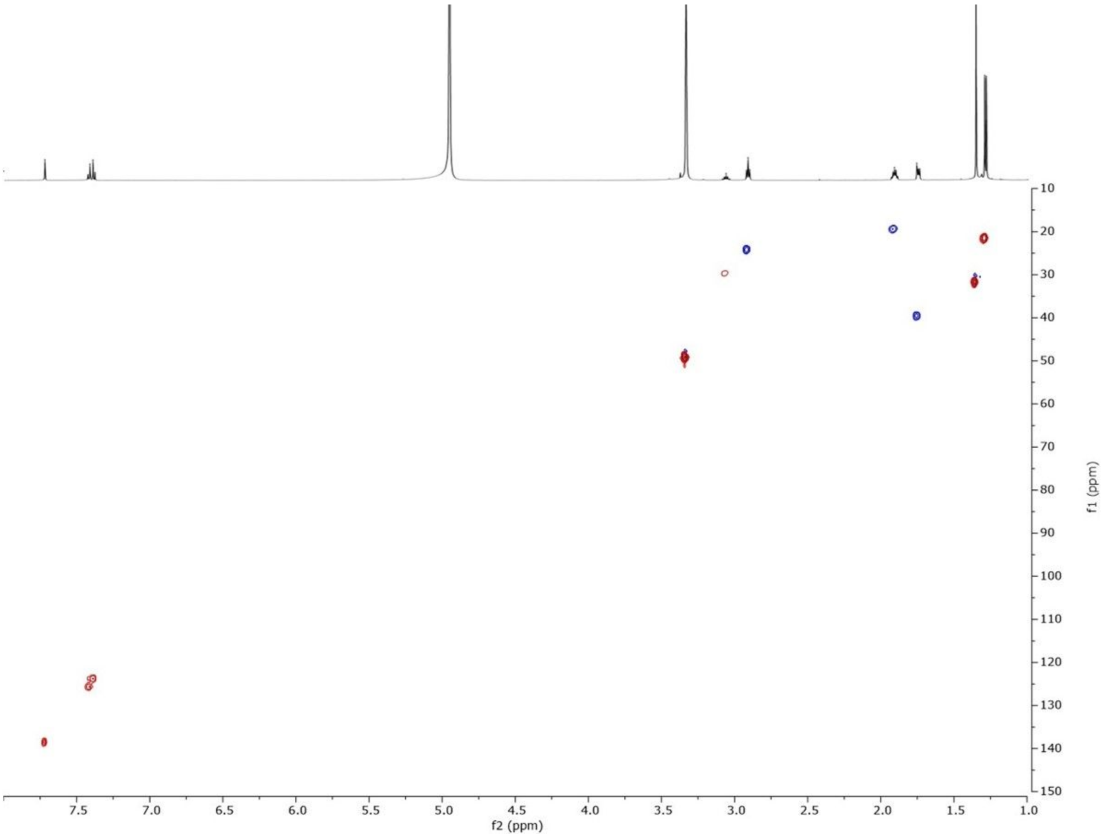

Figure S32. HSQC Spectrum (CD_3OD) of compound **16.**

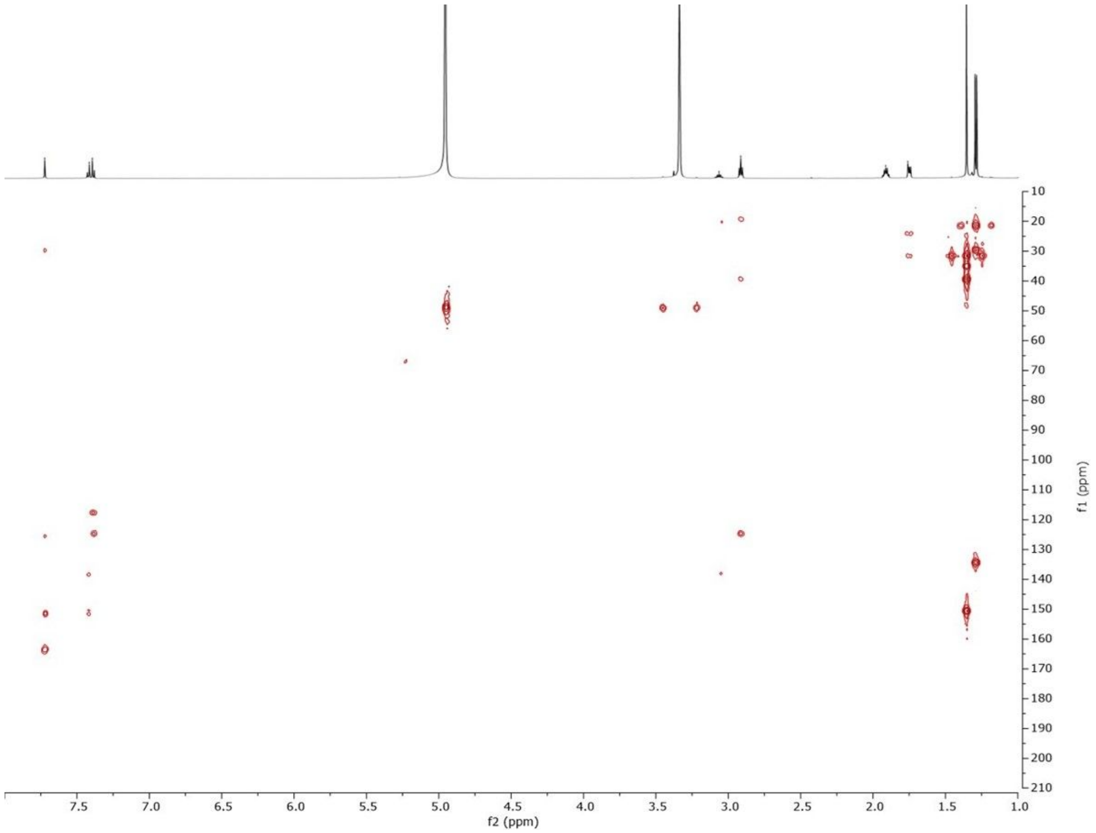

Figure S33. HMBC Spectrum (CD_3OD) of compound **16.**

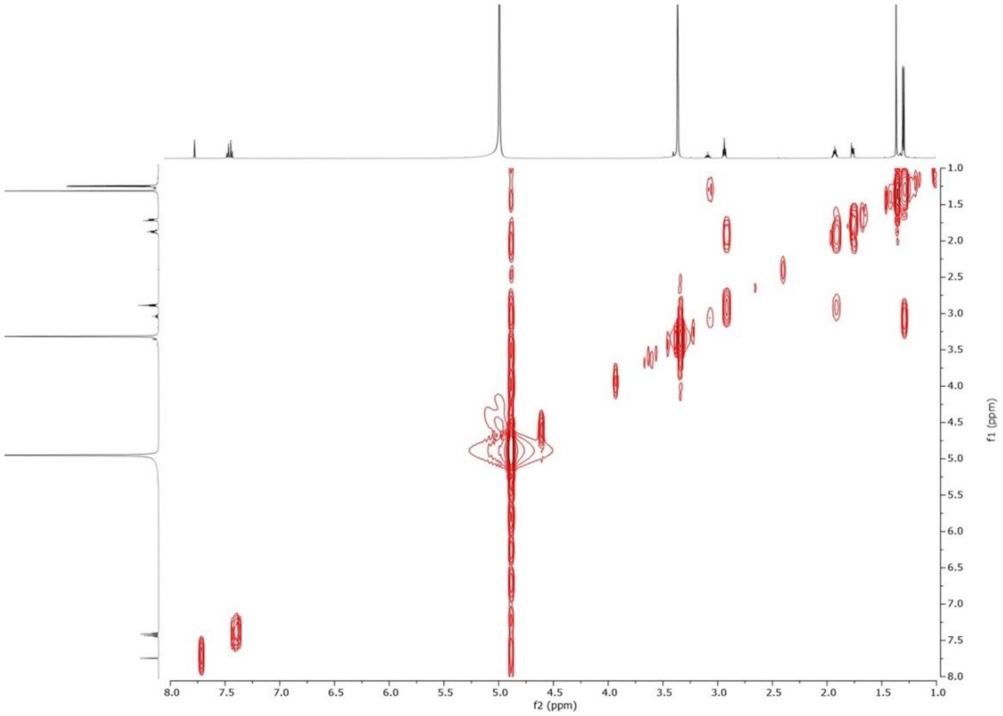

Figure S34. COSY Spectrum (CD_3OD) of compound **16.**

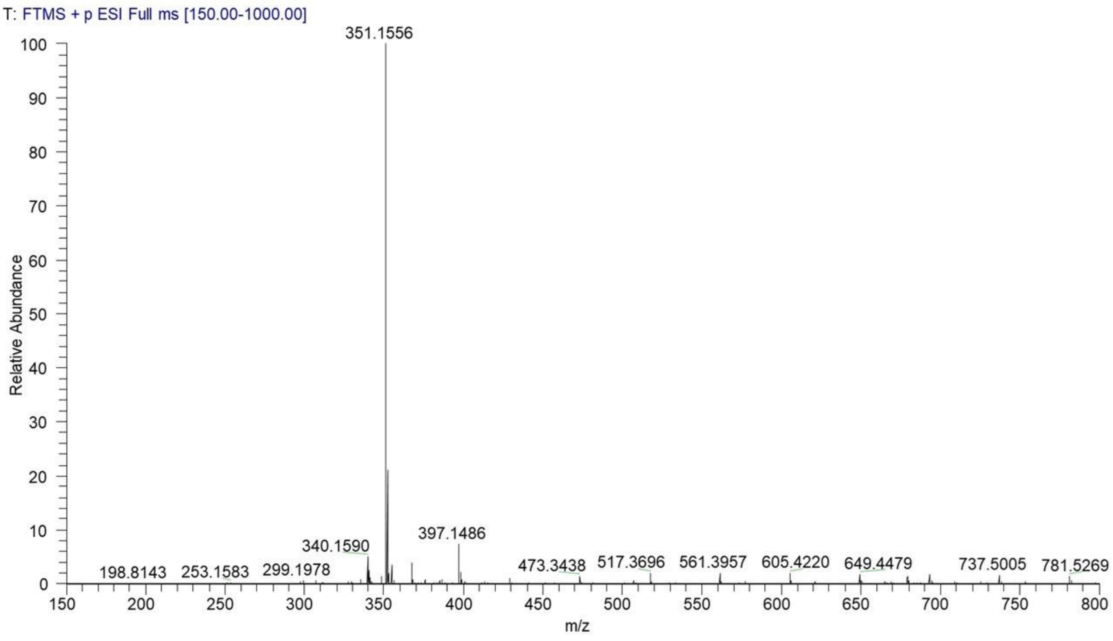

Figure S36. ESI/LTQOrbitrap spectrum of compound **20**, in negative ion mode.

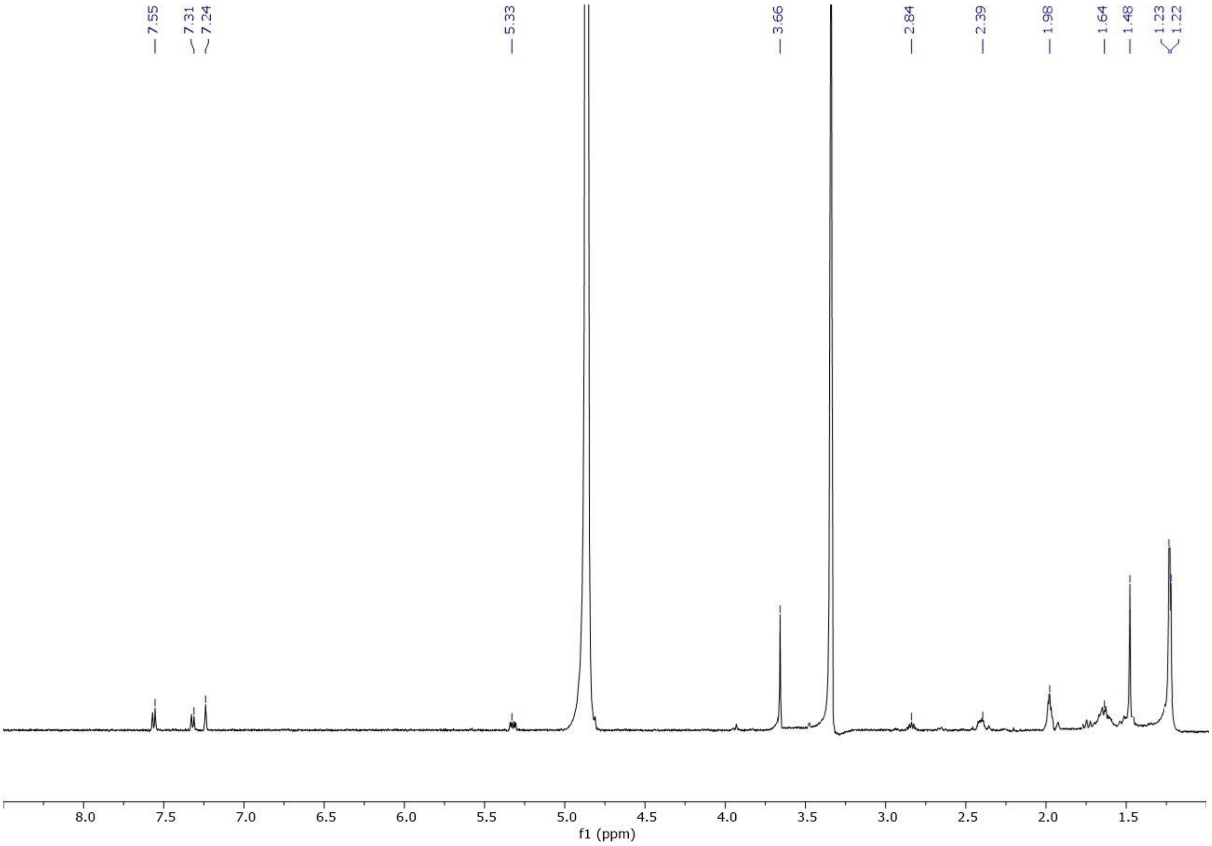

Figure S37. ^{1}H NMR Spectrum (600 MHz, CD_3OD) of compound **20**.

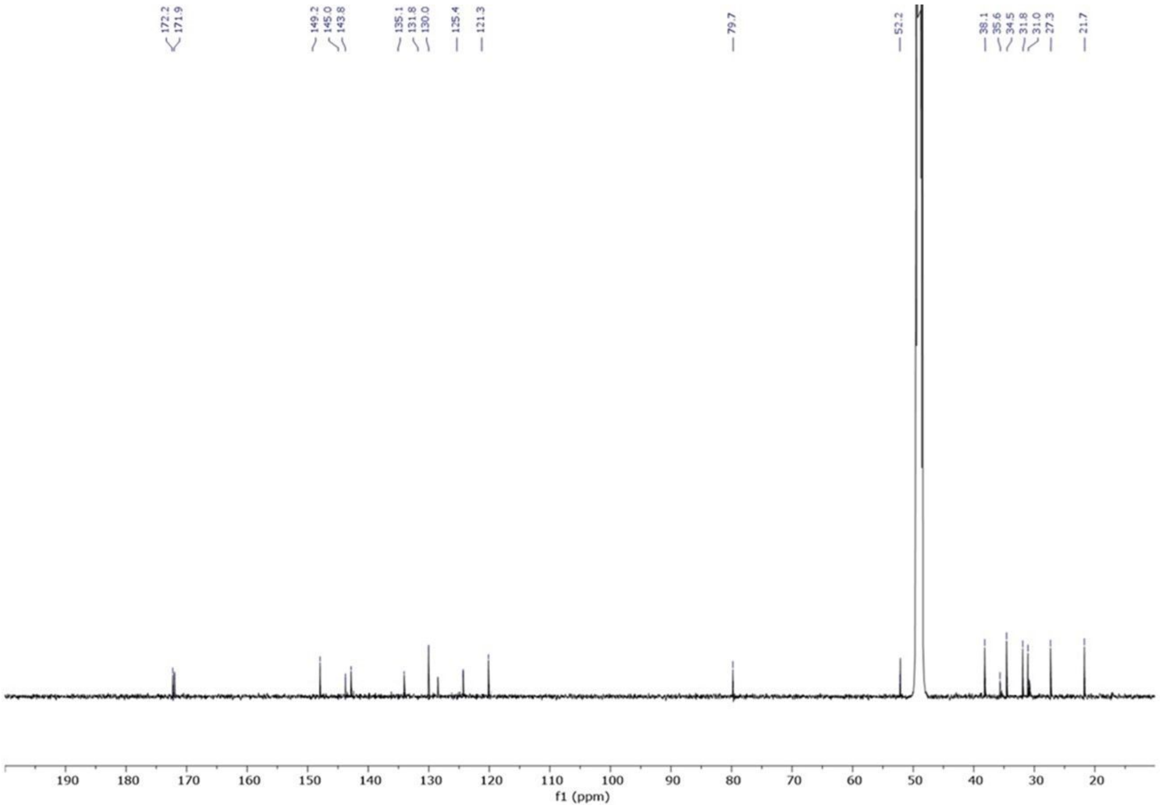

Figure S38. ^{13}C Spectrum (150 MHz, CD_3OD) of compound **20**.

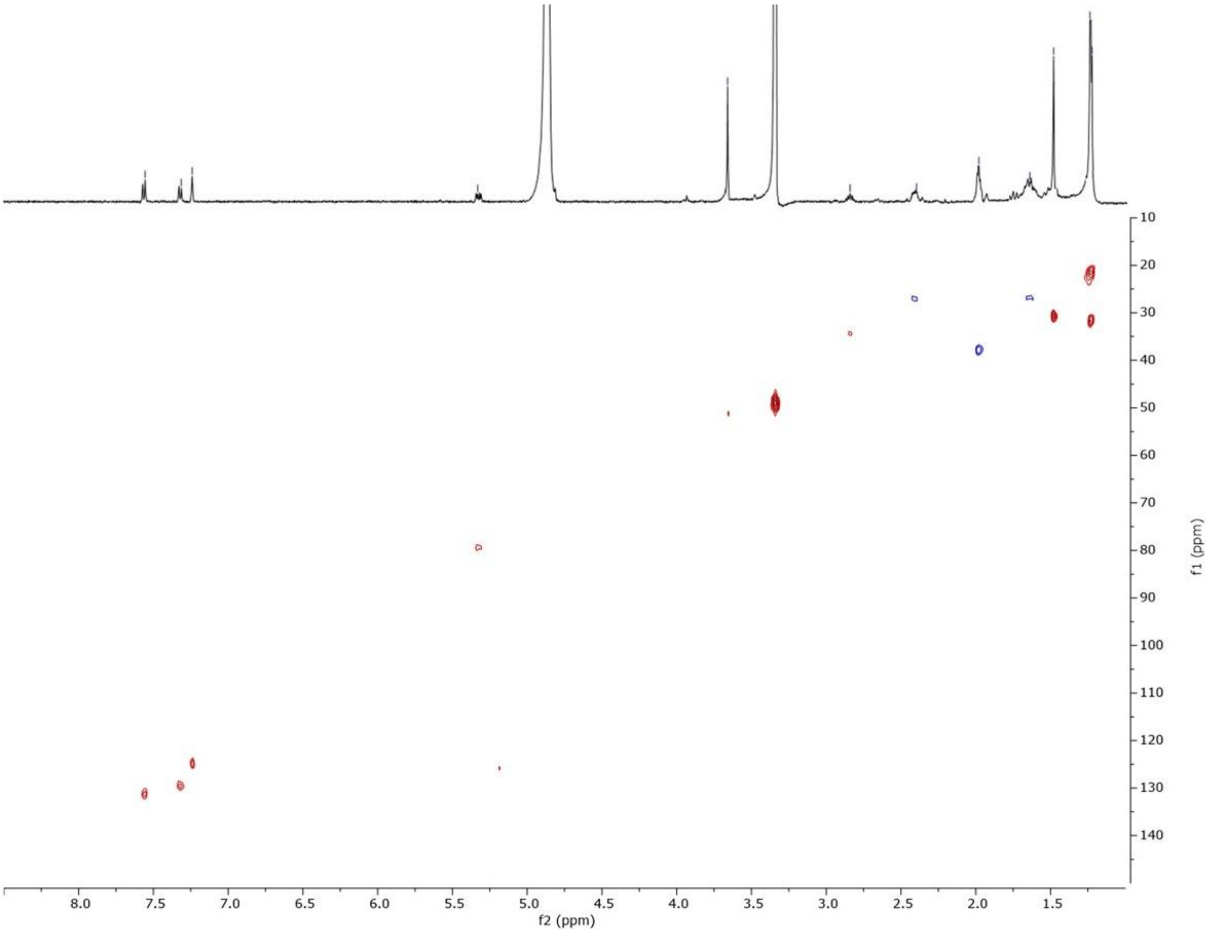

Figure S39. HSQC Spectrum (CD_3OD) of compound **20**.

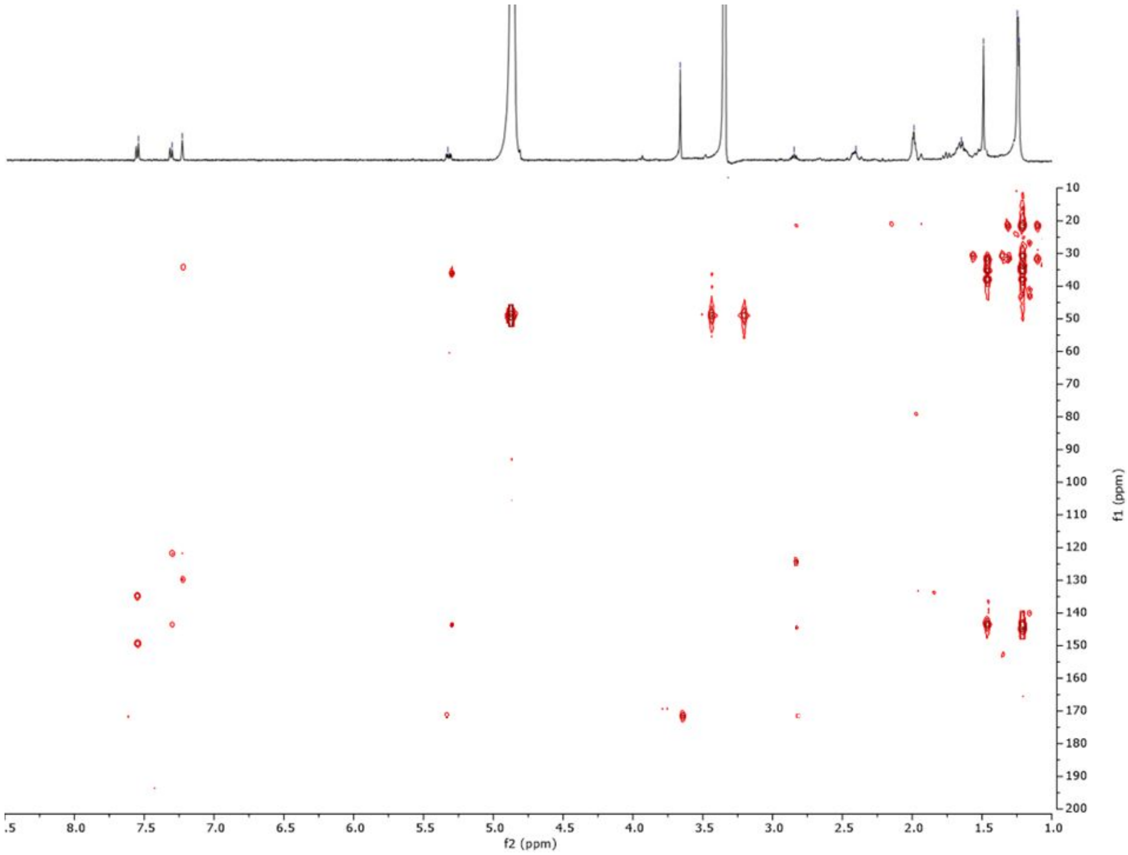

Figure S40. HMBC Spectrum (CD_3OD) of compound **20**.

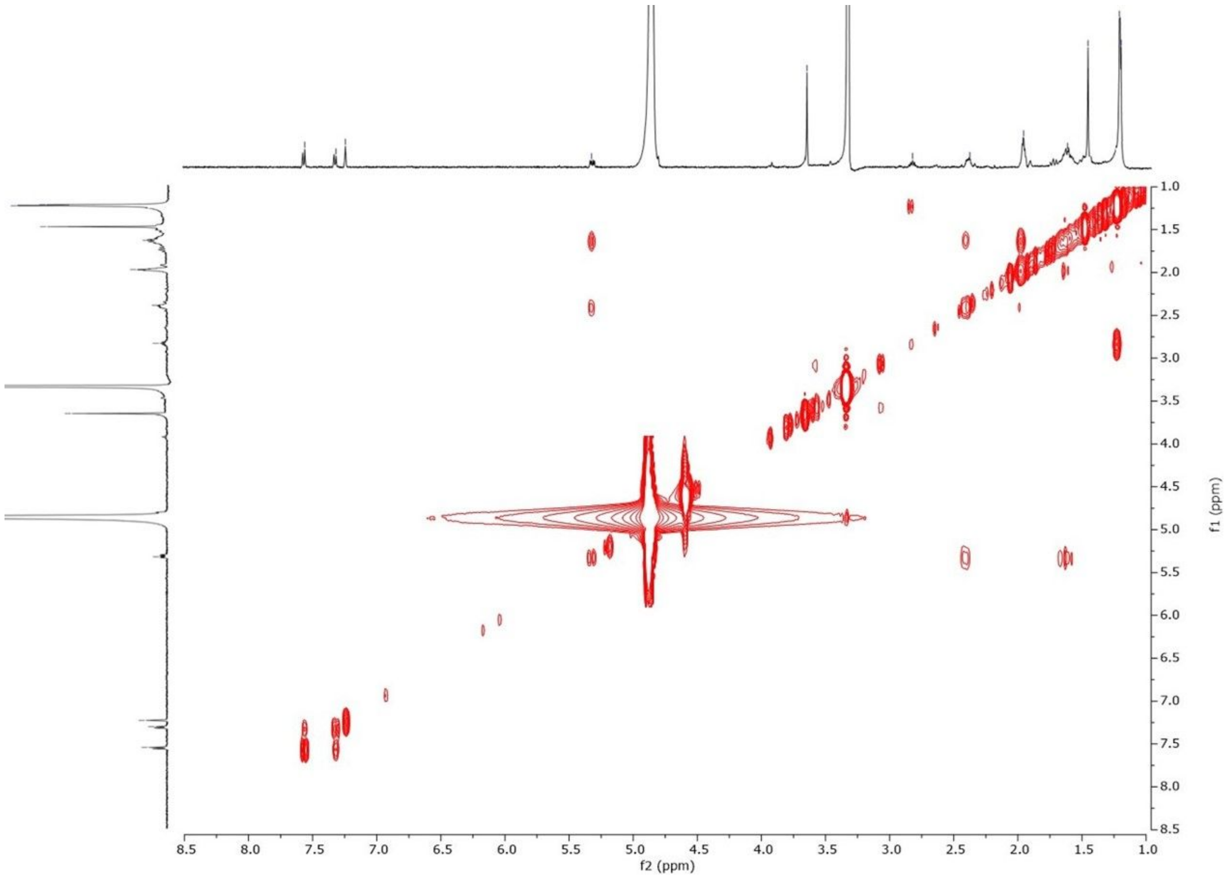

Figure S41. COSY Spectrum (CD_3OD) of compound **20**.
